# Interplay between bile acids, gut microbiota, and the tumor immune microenvironment: mechanistic insights and therapeutic strategies

**DOI:** 10.3389/fimmu.2025.1638352

**Published:** 2025-08-01

**Authors:** Yan Tong, Xiaojun Lou

**Affiliations:** ^1^ Jiaxing University Master Degree Cultivation Base, Zhejiang Chinese Medical University, Hangzhou, Zhejiang, China; ^2^ Department of Gastroenterology, Jiaxing Hospital of Traditional Chinese Medicine, Jiaxing, Zhejiang, China

**Keywords:** bile acids, gut microbiota, tumor immune microenvironment, cancer immunotherapy, immunometabolism

## Abstract

The interplay between the gut microbiota, bile acid (BA) metabolism, and the tumor immune microenvironment (TIME) is a critical and rapidly advancing field in cancer immunology. Microbiota-transformed bile acids act as pivotal signaling molecules. This review systematically dissects how these BAs engage host receptors (e.g., FXR, TGR5, VDR, S1PR2) to influence the differentiation and activity of key innate (macrophages, NK cells, MDSCs, DCs) and adaptive (CD8+ T cells, Tregs, Th1/Th17 cells) immune cell populations within the TIME. We underscore that dysregulation of this microbiota-BA-immune axis, prevalent in gastrointestinal and hepatobiliary cancers, frequently cultivates a pro-inflammatory, immunosuppressive TIME, thereby facilitating tumor immune evasion and progression. In light of this, we examine emerging therapeutic strategies aimed at reprogramming this axis, including pharmacological BA receptor modulation, microbiota-based interventions (e.g., engineered microbes, FMT, dietary strategies), and their synergistic potential with established cancer treatments like immune checkpoint inhibitors. Finally, this review addresses significant challenges in clinical translation, including inherent axis complexity, inter-individual variability, and methodological hurdles. Future directions highlighted include tackling heterogeneity, employing advanced multi-omics, and developing robust biomarkers for precision immuno-oncology. Unraveling this complex immunometabolic network is crucial for identifying novel diagnostic tools and advancing next-generation cancer immunotherapies.

## 1 Introduction

### 1.1 Bile acids: key immuno-metabolic signals sculpted by the gut microbiota

Bile acids (BAs), beyond their traditional role in lipid digestion, are now recognized as pleiotropic signaling molecules crucial for metabolic and immune homeostasis (1, 2). BAs modulate innate and adaptive immunity by influencing immune cell differentiation and activity, and regulating intestinal and systemic inflammation (2–4). The gut microbiota profoundly shapes this signaling by enzymatically transforming primary BAs (e.g., cholic acid, CA; chenodeoxycholic acid, CDCA) into diverse secondary and modified BAs, like deoxycholic acid (DCA) and lithocholic acid (LCA) (1, 5). These microbial BAs, with distinct receptor affinities and activities from their precursors, mediate host-microbiome dialogue and influence host physiology and pathology via specific receptors (1, 2, 6).

### 1.2 The tumor immune microenvironment and dysregulation of the microbiota-bile acid axis in cancer

Before delving into the pathological consequences of a dysregulated microbiota-BA axis, it is important to first establish its fundamental role in maintaining homeostasis. Under physiological conditions, a healthy gut microbiota and an intact intestinal barrier are critical for establishing immune tolerance (7). This is achieved, in part, through the production of beneficial microbial metabolites, such as short-chain fatty acids (SCFAs), which support the function of regulatory T cells (Tregs) and the secretion of anti-inflammatory cytokines like IL-10 (8). This homeostatic state prevents excessive inflammation against commensal microbes and dietary antigens. The disruption of this delicate balance is a pivotal event that can shift the gut from a state of tolerance to one of chronic inflammation, thereby creating a microenvironment conducive to tumorigenesis (9, 10).

The tumor immune microenvironment (TIME), a dynamic ecosystem of cancer cells, stromal cells, ECM, and diverse immune cells, profoundly shapes tumor progression and therapeutic responses (11, 12). The TIME often becomes immunosuppressive, marked by regulatory cell accumulation (e.g., Regulatory T cells (Tregs), Myeloid-derived suppressor cells (MDSCs)), effector cell impairment (e.g., CD8+ T cells, NK cells), and immune checkpoint upregulation, fostering tumor immune evasion (13, 14). Accumulating evidence indicates that the gut microbiota-BA axis, a crucial regulator of host immune homeostasis (15), is often dysregulated in cancer, particularly in gastrointestinal and hepatobiliary malignancies (16, 17). For instance, clinical studies have documented significantly elevated systemic levels of LPS—a hallmark of microbial translocation—in patients with colorectal, pancreatic, and liver cancers, which often correlate with advanced disease stage and poorer prognosis (10, 18). This dysregulation, typically involving gut dysbiosis and altered BA profiles (e.g., increased levels of DCA and LCA), contributes to a pro-carcinogenic milieu by fueling chronic inflammation, promoting an immunosuppressive TIME, and exerting direct pro-tumorigenic effects on cells (16, 19, 20). These pathological effects are often mediated through host receptors that sense altered BA signals within the TIME.

A pivotal mechanism linking gut dysbiosis to pro-tumorigenic inflammation is the translocation of microbial components, most notably lipopolysaccharide (LPS), across a compromised intestinal barrier (21). LPS, an endotoxin derived from the outer membrane of Gram-negative bacteria, is a potent immune stimulant. Its immunostimulatory moiety, lipid A, engages the CD14/TLR4/MD-2 receptor complex on innate immune cells, particularly monocytes and macrophages (21, 22). This engagement triggers a MyD88-dependent signaling cascade that culminates in the activation of the nuclear factor-κB (NF-κB) transcription factor. Once translocated to the nucleus, NF-κB drives the expression of a broad array of pro-inflammatory genes, including those encoding cytokines such as TNF-α, IL-6, and IL-1β (22, 23). This sustained LPS-TLR4-NF-κB signaling axis is instrumental in establishing a chronic inflammatory state within the tumor microenvironment, which is highly conducive to tumorigenesis and immune evasion (21–23).

Beyond TLR-dependent microbial sensing, the NF-κB pathway serves as a critical signaling hub that integrates diverse pro-tumorigenic stimuli within the TIME. Notably, NF-κB can be activated independently of TLRs by inflammatory cytokines, such as Tumor Necrosis Factor-alpha (TNF-α), which are abundant in the tumor microenvironment. Upon binding to its receptor (TNFR), TNF-α initiates a distinct signaling cascade that also converges on the activation of the IKK complex, leading to the liberation of the canonical NF-κB heterodimer, p50/p65 (RelA).

**Figure 1:**
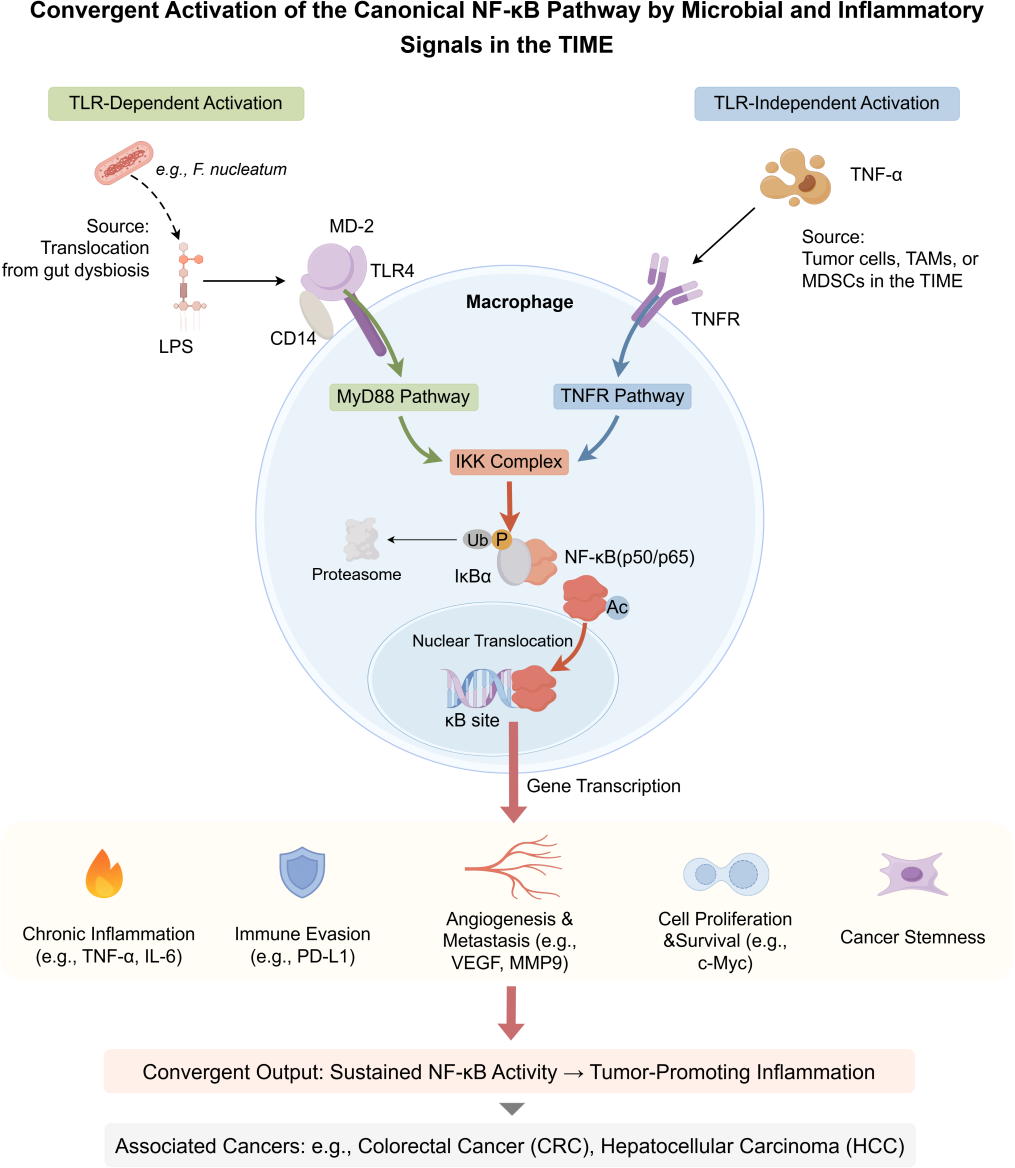
Convergent activation of the canonical NF-κB pathway by microbial and inflammatory signals in the TIME. This schematic illustrates two major upstream pathways that activate canonical NF-κB signaling in macrophages. (Left, green pathway) The TLR-dependent axis is initiated by microbial components such as lipopolysaccharide (LPS), which engage the CD14/MD-2/TLR4 receptor complex. (Right, blue pathway) The TLR-independent axis is triggered by pro-inflammatory cytokines like tumor necrosis factor-alpha (TNF-α), binding to TNFRs expressed in the TIME. Both axes converge on the activation of the IκB kinase (IKK) complex. IKK phosphorylates the inhibitor of NF-κB alpha (IκBα), promoting its ubiquitination and degradation by the proteasome. This releases the active NF-κB heterodimer (p50/p65), which undergoes further post-translational modifications (PTMs), such as phosphorylation and acetylation. The activated NF-κB translocates to the nucleus, binds to κB DNA response elements, and initiates transcription of tumor-promoting genes. Target genes contribute to key hallmarks of cancer: (1) chronic inflammation (e.g., *TNF, IL-6*), (2) immune evasion (e.g., *PD-L1*), (3) angiogenesis and metastasis (e.g., *VEGF, MMP9*), (4) cell proliferation and survival (e.g., *MYC*), and (5) cancer stemness. Sustained NF-κB activity thus mechanistically links microbial cues and chronic inflammation to cancers such as colorectal cancer (CRC) and hepatocellular carcinoma (HCC). While macrophages are the central model in this schematic, similar mechanisms operate in other myeloid cells within the TIME. (P, phosphorylation; Ub, ubiquitination; Ac, acetylation). By Figdraw.

Once in the nucleus, the activated NF-κB heterodimer initiates transcription of a broad array of target genes underpinning multiple cancer hallmarks. These include pro-inflammatory cytokines (e.g., TNF, IL-6) that sustain chronic inflammation (22, 24); immune checkpoint molecules (e.g., PD-L1) that promote immune evasion (22); angiogenic and metastatic effectors (e.g., VEGF, MMP9) (24, 25); and regulators of proliferation and survival (e.g., MYC, BCL2) (22, 26). Crucially, this multifaceted role extends to the regulation of cancer stemness (27). Within the CSC niche, NF-κB activation is pivotal for maintaining key properties such as self-renewal and therapeutic resistance, thereby driving tumor relapse and progression. Through this diverse array of outputs, the sustained activation of NF-κB, driven by either microbial or inflammatory signals, serves as a master regulatory link to tumorigenesis in contexts such as colorectal cancer (CRC) and hepatocellular carcinoma (HCC) (22, 24). These converging pathways that orchestrate NF-κB activation are summarized in **Figure 1**.

### 1.3 Bile acid receptors, rationale, and scope of this review

The immunomodulatory effects of BAs are primarily transduced through a repertoire of host receptors, including nuclear receptors like the Farnesoid X receptor (FXR) and Vitamin D receptor (VDR), and G protein-coupled receptors such as Takeda G protein-coupled receptor 5 (TGR5) and Sphingosine-1-Phosphate Receptor 2 (S1PR2) (2, 28, 29). These receptors, expressed on various immune and non-immune cells within the TIME, sense the altered BA pool and translate these microbial metabolic signals into cellular responses. Understanding the microbiota-BA-TIME interplay is crucial for deciphering tumor immune evasion and developing novel therapies, given BAs’ roles as microbiota-shaped signals and the TIME’s impact on cancer. Therefore, This review dissects how microbiota-derived BAs, via these receptors, modulate TIME immune cells. We then explore therapeutic strategies targeting this axis, current challenges, and future perspectives for translating insights into therapies.

## 2 Bile acid-mediated regulation of immune cell populations and functions within the TIME

**Figure 2:**
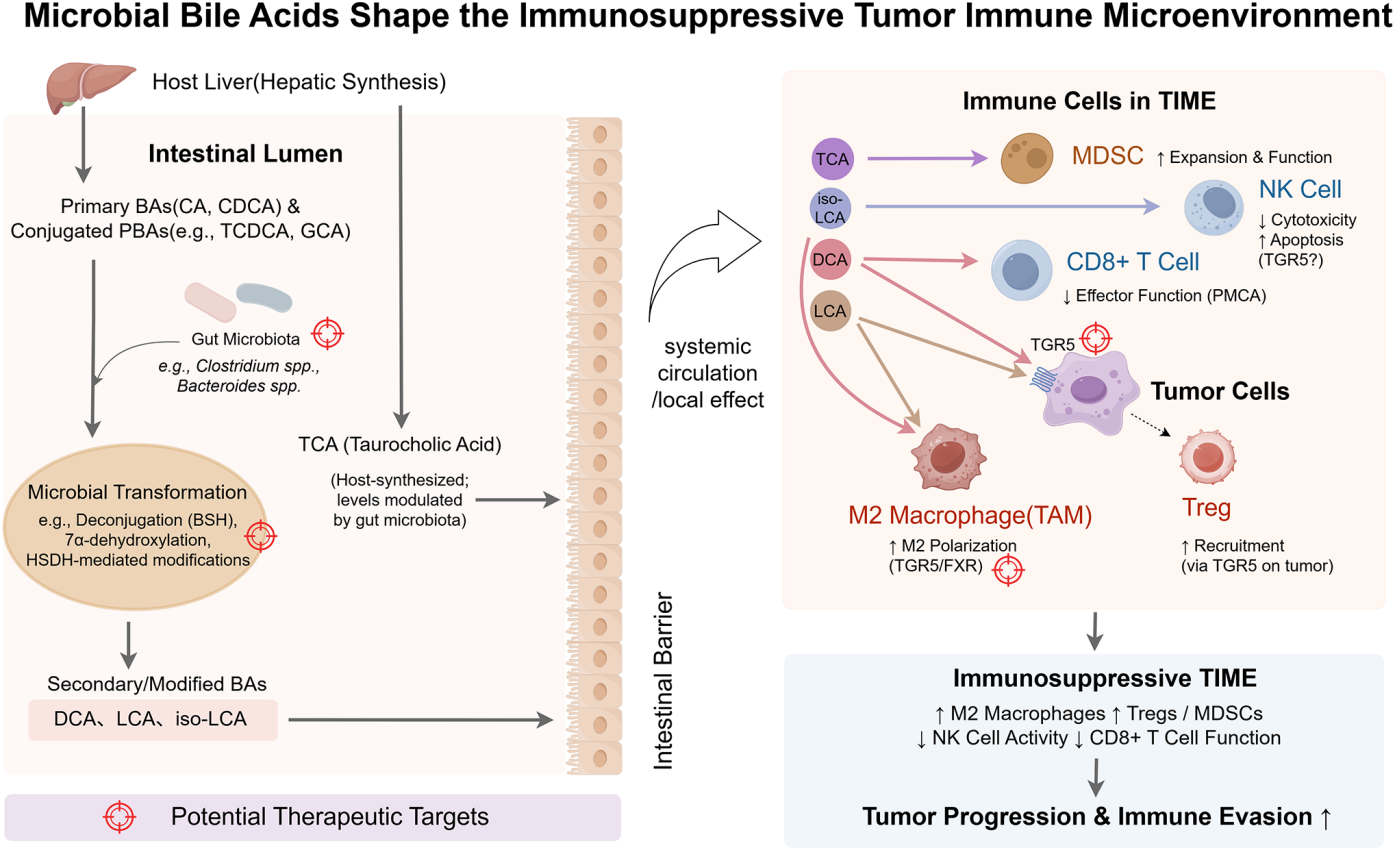
Microbial bile acids shape the immunosuppressive tumor immune microenvironment (TIME). This schematic illustrates how the gut microbiota transforms host-derived bile acids (BAs) into immunomodulatory metabolites that shape an immunosuppressive tumor immune microenvironment (TIME). Primary BAs (PBAs; e.g., cholic acid (CA), chenodeoxycholic acid (CDCA)) and conjugated BAs (e.g., taurocholic acid (TCA)) are synthesized in the liver and secreted into the intestine. There, microbial species (e.g., Clostridium, Bacteroides) convert them via bile salt hydrolase (BSH), 7α-dehydroxylation, and related enzymatic reactions into secondary BAs (SBAs), including deoxycholic acid (DCA), lithocholic acid (LCA), and isolithocholic acid (iso-LCA). These BAs cross the intestinal barrier and enter the TIME via systemic circulation or local signaling. Within the TIME, specific BAs bind to host receptors on immune or tumor cells to mediate immunosuppressive effects. DCA promotes M2 macrophage polarization and impairs CD8^+^ T cell function; tumor-cell Takeda G-protein-coupled receptor 5 (TGR5) signaling enhances regulatory T cell (Treg) recruitment. LCA similarly supports M2 polarization and Treg accumulation. iso-LCA suppresses natural killer (NK) cell cytotoxicity and induces apoptosis. TCA promotes the expansion of myeloid-derived suppressor cells (MDSCs). Color-coded immune cell types reflect functional states: immunosuppressive cells (M2 macrophages, Tregs, MDSCs) appear in warm tones; effector cells (CD8^+^ T cells, NK cells) are shown in cooler tones, though functionally impaired. Tumor cells appear in purple. Color-coded arrows represent BA-specific signaling pathways, and target icons denote therapeutic intervention points (e.g., microbial enzymes, BA receptors, microbiota composition). By Figdraw.

Bile acids (BAs) regulate the TIME through a spectrum of diverse mechanisms. While this review primarily focuses on the well-characterized receptor-mediated signaling pathways, it is crucial to first acknowledge that at high pathophysiological concentrations, certain hydrophobic secondary BAs, such as deoxycholic acid (DCA), can exert direct cytotoxic effects on cancer cells, which may complement their immunomodulatory roles. *In vitro* studies have demonstrated that DCA can induce apoptosis in colon adenocarcinoma cells, a process mechanistically linked to the generation of oxidative stress and subsequent mitochondrial dysfunction (30). Although some studies have investigated the direct impact of DCA on membrane physical properties, the prevailing evidence suggests that its primary cytotoxic actions are mediated through intracellular stress pathways rather than a direct, detergent-like disruption of the plasma membrane (31). These findings highlight a direct, non-receptor-mediated mechanism by which BAs can influence cancer cell viability. Elucidating how these direct cytotoxic effects intersect with BA-receptor signaling within the complex cellular milieu of the TIME remains a key area for future investigation. **Figure 2** illustrates how microbiota-derived bile acids modulate immune cells to foster an immunosuppressive TIME—a central theme of this section.

### 2.1 Modulation of innate immune cell populations by bile acids

#### 2.1.1 Macrophages: polarization, phagocytosis, and cytokine production

Macrophages are key targets of bile acids (BAs) in the TIME, given their immune plasticity and roles in inflammation and tumor progression (2, 3). BA-modulated macrophage functions include M1/M2 polarization, phagocytosis, and cytokine production, largely shaped by the microbiota-regulated BA milieu.

Macrophage BA responses are mediated by receptors such as farnesoid X receptor (FXR), Takeda G protein-coupled receptor 5 (TGR5), vitamin D receptor (VDR), liver X receptor alpha (LXRα), and peroxisome proliferator-activated receptor gamma (PPARγ) (2, 3, 32–35). FXR activation by primary BAs like chenodeoxycholic acid (CDCA) or secondary BAs like ursodeoxycholic acid (UDCA) suppresses pro-inflammatory cytokines (e.g., TNF-α, IL-6) and promotes M2-like features via inhibition of NF-κB and NLRP3 pathways. TGR5, activated by lithocholic acid (LCA) and deoxycholic acid (DCA), reduces inflammatory signaling through cAMP pathways and enhances IL-10 expression. VDR and PPARγ, responsive to LCA and nor-ursodeoxycholic acid (norUDCA), respectively, also support anti-inflammatory M2 polarization.

However, BA effects are context-dependent. DCA promotes M2 polarization of tumor-associated macrophages (TAMs) in the TIME, enhancing tumor growth and immune evasion (4, 12, 36). Certain hydrophobic BAs, such as DCA and LCA, may paradoxically activate the NLRP3 inflammasome under specific conditions, inducing IL-1β production despite their typical inhibitory roles (33).

BAs also affect macrophage recruitment. LCA, DCA, and hyodeoxycholic acid (HDCA) suppress chemokines like CCL2 and CCL8, with LXRα activation implicated in this effect for DCA and HDCA (37). Conjugated BAs, including taurocholic acid (TCA), taurochenodeoxycholic acid (TCDCA), and tauroursodeoxycholic acid (TUDCA), inhibit IL-6 expression in macrophage lines (38). Their direct effects on phagocytosis are less defined (2, 39).

In sum, BAs—especially those shaped by microbial metabolism—modulate macrophages through multiple nuclear and membrane receptors, balancing anti-inflammatory responses and pro-tumor M2 TAM expansion, underscoring their dual role in the TIME.

#### 2.1.2 Natural killer cells: direct inhibition of cytotoxicity, activation, and survival by iso-LCA

Natural killer (NK) cells are critical components of the innate immune system, playing a vital role in tumor immunosurveillance and elimination within the TIME through direct cytotoxicity and cytokine production (40, 41). However, NK cell function is often suppressed in the established tumor microenvironment (42, 43). Emerging evidence indicates that specific bile acids (BAs), particularly those modulated by the gut microbiota, can directly impact NK cell activity and survival, potentially contributing to tumor immune evasion.

Specifically, recent work by Wei et al. (2025) elucidated a key pathway involving the secondary BA isolithocholic acid (iso-LCA). They found that loss of the hepatic BA-metabolizing enzyme AKR1D1 leads to gut dysbiosis, characterized by an increased proportion of Bacteroidetes ovatus. This specific bacterium metabolizes the primary BA chenodeoxycholic acid (CDCA) into iso-LCA, which subsequently accumulates in the liver (44). Crucially, this accumulated iso-LCA was found to directly suppress NK cell cytotoxicity, evidenced by reduced secretion of key effector cytokines interferon-γ (IFN-γ) and tumor necrosis factor-α (TNF-α). This suppression was mediated by inhibition of p-CREB1 phosphorylation in NK cells, a key regulator of cytokine expression (44, 45). Furthermore, iso-LCA treatment induced apoptosis in NK cells, thereby compromising their survival within the microenvironment (44). The pro-tumorigenic effect of iso-LCA observed in their models was demonstrated to be dependent on NK cells, as NK cell depletion abrogated the difference in tumor growth between control and iso-LCA-treated mice (44). Molecular docking simulations and the antagonistic effect of spironolactone (SPI) in their study suggested that the Takeda G protein-coupled receptor 5 (TGR5/GPBAR1) might mediate these effects of iso-LCA on NK cells, although direct functional validation is pending (44). This pathway highlights a direct mechanism by which microbiota-derived BA metabolites can impair innate anti-tumor immunity by suppressing NK cell function and survival.

#### 2.1.3 Myeloid-derived suppressor cells: bile acid-mediated regulation

Myeloid-derived suppressor cells (MDSCs), a heterogeneous population of immature myeloid cells with strong immunosuppressive activity, play a pivotal role in tumor immune evasion and metastasis (46, 47). Microbiota-derived BAs promote the expansion and immunosuppressive programming of MDSCs in the TIME.

Among these, conjugated and secondary BAs, particularly taurocholic acid (TCA) and deoxycholic acid (DCA), exert notable effects on MDSCs. In a colorectal cancer (CRC) lung metastasis model, gut dysbiosis-induced TCA accumulation promoted M-MDSC expansion and immunosuppressive function by enhancing glycolysis and epigenetically stabilizing PD-L1 expression via H3K4 mono-methylation, with potential involvement of the Farnesoid X Receptor (FXR) (48). Likewise, DCA, in synergy with bacterial lipopolysaccharide (LPS), activates colonic macrophages through the LPS/HMGB1/NF-κB pathway to produce chemokines (e.g., CCL2) and cytokines (e.g., TNF-α), which facilitated the recruitment and activation of both G-MDSCs and M-MDSCs (49).

Conversely, targeting BA metabolism offers a promising strategy to reduce MDSC-mediated immunosuppression. In a MAFLD-related hepatocellular carcinoma (HCC) model, supplementation with *Akkermansia muciniphila* reduced circulating levels of TCA and DCA, leading to decreased hepatic M-MDSCs and enhanced T cell infiltration and PD1 response (50). Furthermore, in colitis-associated cancer (CAC), dysbiotic gut microbiota was shown to drive MDSC accumulation and functional reprogramming, potentially through BA composition shifts and their interaction with infiltrating bacteria (51).

Collectively, these findings highlight microbiota-derived BAs as critical regulators of MDSC biology in the TIME. Elucidating these BA–MDSC interaction pathways may unveil novel therapeutic targets to overcome immune suppression and tumor progression.

#### 2.1.4 Dendritic cells: maturation, antigen presentation, and cytokine release

Dendritic cells (DCs), as key antigen-presenting cells (APCs), orchestrate anti-tumor responses in the TIME (52, 53). Their maturation, antigen presentation, and cytokine profiles critically shape T cell activation. Bile acids (BAs), especially those derived from gut microbiota, exert diverse effects on DCs depending on BA type, DC subset, and context.

Microbial metabolites like isodeoxycholic acid (isoDCA), converted from deoxycholic acid (DCA) by bacteria such as Eggerthella lenta and Clostridium scindens (54, 55), promote a tolerogenic phenotype in DCs, potentially through functional antagonism of the FXR receptor. IsoDCA downregulates MHC II and antigen processing genes (e.g., Ciita), suppresses pro-inflammatory cytokines (e.g., TNF-α, IL-6), and promotes peripheral Treg differentiation, fostering immune tolerance (55).

Similarly, secondary BAs like LCA and DCA suppress DC cytokine secretion (e.g., IL-12, IL-6) and co-stimulatory molecule expression through TGR5 activation, promoting tolerogenic phenotypes (56, 57).

Nonetheless, BA effects vary by context. For instance, DCA can activate plasmacytoid DCs to induce type I IFNs via TLR7/MyD88 signaling (58, 59). In therapies such as low-dose intestinal irradiation (ILDR) with PD-L1 blockade, BAs like DCA and UDCA enhance DC antigen presentation, contributing to improved immunotherapy outcomes (60).

Other BAs like TUDCA modulate the DC microenvironment indirectly, while 3-oxoLCA and isoLCA inhibit Th17 cells, synergizing with isoDCA-driven Treg promotion (54, 61, 62).

Thus, the gut BA pool regulates DC function via FXR, TGR5, and Toll-like receptors (TLRs), driving either tolerance or activation depending on context. This BA–DC axis presents a promising therapeutic target for reshaping the TIME.

### 2.2 Modulation of adaptive immune cells by bile acids

Beyond their impact on innate immunity, bile acids (BAs) also exert profound and multifaceted regulatory effects on adaptive immune cell populations, critically shaping T cell differentiation, effector functions, and memory responses within the TIME. The following sections will detail the influence of BAs on key adaptive lymphocytes, including CD8+ T cells, regulatory T cells, and T helper subsets.

#### 2.2.1 CD8+ T cells: modulation by bile acids in effector function, exhaustion, and ICI response

Cytotoxic CD8^+^ T lymphocytes are central effectors in the TIME, and their function strongly influences anti-tumor immunity and immune checkpoint inhibitor (ICI) efficacy (63, 64). Growing evidence highlights the gut microbiota and its bile acid (BA) metabolites as key regulators of CD8^+^ T cell activity and ICI responsiveness (65, 66).

Certain BAs can bidirectionally regulate CD8^+^ T cell effector functions directly. Deoxycholic acid (DCA), a secondary BA, inhibits the plasma membrane Ca²^+^ ATPase (PMCA), suppressing Ca²^+^-NFAT2 signaling and reducing cytotoxic molecules such as IFN-γ, TNF-α, and granzyme B (GzmB), potentially impairing ICI efficacy when intratumoral DCA is high (64, 67). In contrast, taurolithocholic acid (TLCA) promotes CD8^+^ T cell activation and cytokine production, and has been associated with improved ICI response in non-small cell lung cancer, possibly via enhanced memory CD8^+^ T cell formation (68). Higher BA metabolism in hepatocellular carcinoma (HCC) correlates with increased CD8^+^ T cell infiltration and favorable outcomes (20), though this relationship is context-dependent. For example, FKBP-5 deletion, which reduces intestinal BAs, paradoxically enhances CD8^+^ T cell infiltration and limits HCC progression (69).

The microbiota shapes the BA pool and influences CD8^+^ T cell function. Akkermansia muciniphila supplementation improves PD-1 blockade efficacy in MAFLD-related HCC models by restoring intestinal barrier integrity, lowering LPS and BA metabolite translocation, and reducing suppressive cells (MDSCs, M2 macrophages), thereby enhancing CD8^+^ T cell infiltration (50). Similarly, intestinal low-dose irradiation (ILDR) augments PD-L1 therapy via microbiota and BA changes—such as increases in Christensenella minuta and favorable BAs (DCA, UDCA)—that enhance dendritic cell antigen presentation and CD8^+^ T cell activation (60). Microbial dysbiosis can also promote CD8^+^ T cell exhaustion and impair anti-tumor immunity (63).

While this review emphasizes BAs, other gut microbiota-derived metabolites (e.g., inosine, short-chain fatty acids) also regulate CD8^+^ T cells through distinct pathways (63, 65, 66). Whether BAs directly reprogram CD8^+^ T cell metabolic circuits remains under investigation (64).

In summary, BA metabolites from the gut microbiota critically shape CD8^+^ T cell-mediated anti-tumor immunity and ICI responsiveness. Modulating the microbiota–BA axis offers a promising avenue to enhance therapeutic outcomes via CD8^+^ T cell reprogramming.

#### 2.2.2 Regulatory T cells: differentiation, stability, and suppressive capacity

Bile acids (BAs), extensively modified by the gut microbiota, critically regulate regulatory T cell (Treg) differentiation, stability, and suppressive functions. Tregs maintain immune tolerance, and their dysregulation in the TIME undermines anti-tumor immunity (63).

Microbial BA metabolism generates secondary or modified BAs that promote Treg development. IsoalloLCA, a lithocholic acid (LCA) derivative, enhances peripheral Treg (pTreg) differentiation via FoxP3 upregulation in a TGF-β and CNS3-dependent manner through mitochondrial Reactive Oxygen Species (ROS), independent of the Vitamin D Receptor (VDR) (70). In contrast, LCA and 3-oxoLCA act via VDR to sustain RORγt^+^ Tregs in the colon (71, 72). Other BAs, including isoDCA and omega-muricholic acid (ω-MCA), indirectly promote pTregs by modulating dendritic cells (DCs), with FXR potentially mediating isoDCA’s effects (55). These findings highlight the specificity of BA-receptor interactions in shaping Treg responses.

Treg stability is less directly studied but may be supported by VDR-mediated maintenance of RORγt^+^ colonic Tregs (71) and reduced IL-6 production (a destabilizing cytokine) via conjugated BAs like TCA, TCDCA, and TUDCA (38, 73). Tregs generated with isoalloLCA show sustained FOXP3 expression after transfer, further suggesting enhanced stability (70). Fecal microbiota transplantation (FMT), which restores microbial and BA profiles, also supports Treg persistence (73).

The impact of BAs on Treg suppressive capacity is remarkably context-dependent, illustrating the dual nature of this regulatory axis. In intestinal homeostasis, BA-induced RORγt+ Tregs exhibit potent anti-inflammatory functions, ameliorating colitis (71). IsoalloLCA-induced Tregs similarly suppress colitis *in vivo* in adoptive transfer models (70). Their role is also essential for the efficacy of FMT in Clostridioides difficile infection (CDI), where Tregs help control inflammation and support beneficial microbial (including BA-metabolizing) engraftment (72, 73). However, within the TIME, this axis can be subverted to promote immunosuppression. For instance, in colorectal cancer (CRC), microbially produced secondary BAs like deoxycholic acid (DCA) and LCA can activate TGR5 on tumor cells, leading to CCL28-mediated recruitment of immunosuppressive Tregs into the tumor, thereby fostering tumor progression (74). This is consistent with observations that specific intratumoral BA profiles in CRC correlate with high Treg abundance and poor patient outcomes (75), and that BA dysregulation in hepatocellular carcinoma (HCC) is linked to Th17/Treg imbalances potentially affecting immunotherapy responses (76).

Collectively, the gut microbiota generates a diverse BA repertoire that, through host receptors such as VDR, FXR, and TGR5 on Tregs, DCs, or tumor cells, profoundly regulates Treg differentiation, stability, and suppressive functions. This microbiota–BA–Treg axis underlies immune homeostasis and TIME modulation, offering mechanistic insights and therapeutic potential.

#### 2.2.3 Modulation of T helper 1 and T helper 17 cells

The gut microbiota-bile acid (BA) axis also crucially differentially regulates T helper 1 (Th1) and T helper 17 (Th17) cells, key players influencing the TIME.

In the case of Th17 cells, specific secondary BAs (SBAs) act as direct regulators by targeting the key transcription factor RORγt. Lithocholic acid (LCA) derivatives, including 3-oxoLCA (70, 77) and lithocholic acid 3-sulfate (LCA-3-S (78), function as RORγt antagonists, binding the receptor to suppress its activity and inhibit Th17 differentiation and IL-17 production. IsoalloLCA, another LCA isomer, primarily induces peripheral Treg (pTreg) differentiation via mitochondrial ROS generation and epigenetic regulation dependent on the Foxp3 CNS3 enhancer (70). While its main role is in promoting Treg differentiation, isoalloLCA also exerts some *in vitro* inhibitory effects on Th17 cells, independent of RORγt modulation (70), thereby indirectly influencing the Th17/Treg balance.

Microbiota-modulating interventions reinforce this connection. For example, *Bifidobacterium pseudocatenulatum* increases SBAs such as LCA and DCA, suppressing Th1/Th17 responses (79), while ginsenoside Rk3 reduces IL-17 production by shifting BA profiles—an effect primarily observed on ILC3s, another IL-17 source (80). Additionally, activation of FXR signaling in macrophages suppresses IL-23 production, thereby limiting Th17 maintenance and function (81). Thus, targeting BA-driven Th17 regulation presents a potential strategy to mitigate pro-tumorigenic effects and enhance responses to immune checkpoint inhibitors (ICIs) (76).

Beyond Th17 suppression, the microbiota–BA axis also influences Th1 cells in a context-dependent manner. LCA inhibits Th1 differentiation in T cell lines, likely via Vitamin D Receptor (VDR) signaling, as siRNA knockdown of VDR attenuates this effect (77). FXR activation in macrophages may also indirectly reduce Th1 responses, as shown in models where FXR agonism diminished inflammation and IFN-γ^+^ T cell populations (81). In contrast, novel microbial amino acid-conjugated BAs (e.g., Trp-CDCA), identified through reverse metabolomics, robustly enhanced IFN-γ production in CD4^+^ T cell assays, suggesting a Th1-promoting role for select microbial BA metabolites that contrasts with LCA’s inhibitory effects (82). The specific receptors mediating these effects remain to be elucidated.

The microbiota–BA axis also intersects with T cell function via stress-response pathways, particularly in the BA-rich ileum. The xenobiotic transporter Mdr1 (encoded by ABCB1 in humans, Abcb1a/b in mice) plays a key protective role. Effector T cells (Teff cells) upregulate Mdr1 upon ileal entry. In its absence, Teff cells become sensitive to conjugated BAs (CBAs), leading to oxidative stress and elevated production of pro-inflammatory cytokines like IFN-γ and TNF-α. This compromises Teff survival and homeostasis within the ileal microenvironment (83), underscoring Mdr1’s essential role in maintaining Teff integrity under high BA conditions.

In summary, the microbiota–BA axis utilizes a range of BA species—including LCA derivatives (3-oxoLCA, LCA-3-S), isomers (isoalloLCA), amino acid conjugates (Trp-CDCA), and CBAs—and engages host pathways such as RORγt, VDR, FXR, Mdr1, and mitoROS to differentially regulate Th1 and Th17 cells. It also supports Teff homeostasis in specific intestinal niches, providing a complex immunometabolic layer of control that shapes the TIME.

### 2.3 Key bile acid-sensing receptors modulating the TIME

#### 2.3.1 Farnesoid X receptor: transcriptional control linking metabolism and immunity in the TIME

The farnesoid X receptor (FXR, encoded by NR1H4), a nuclear receptor superfamily member, is highly expressed in enterohepatic tissues and various immune cells (e.g., macrophages, DCs, T cells) (28, 84). Functioning as a ligand-activated transcription factor, FXR primarily binds unconjugated bile acids (BAs) like chenodeoxycholic acid (CDCA), forms a heterodimer with the retinoid X receptor (RXR), and translocates to the nucleus. There, it binds to FXR response elements (FXREs) in target gene promoters or enhancers, modulating gene transcription (84, 85). Through this mechanism, FXR orchestrates BA homeostasis, lipid and glucose metabolism, and immune regulation (84, 86). Notably, FXR expression can be induced by immune signals like the TLR9-MyD88-IRF7 pathway, linking microbial sensing to its regulatory functions (87). The gut microbiota shapes a diverse pool of BAs, including FXR agonists (e.g., CDCA) and antagonists or modulators (e.g., taurine-β-muricholic acid (T-βMCA) in mice, glycoursodeoxycholic acid (GUDCA) in humans, isoDCA), adding regulatory complexity (55, 88, 89).

Functionally, FXR-mediated transcription often promotes anti-inflammatory effects and supports immune homeostasis. In myeloid cells such as macrophages and DCs, FXR activation typically represses pro-inflammatory gene expression (often via NF-κB inhibition) and can promote M2 polarization (32, 81). Specific microbial BAs, like isoDCA acting as an FXR antagonist in DCs, downregulate antigen presentation and inflammatory signaling genes, thereby favoring regulatory T cell (Treg) differentiation (55).

However, FXR’s transcriptional impact is context-dependent. In graft-versus-host disease (GVHD) models, inflammation-induced loss of microbial FXR antagonists or pharmacological FXR agonism exacerbated disease. Conversely, T cell-specific FXR deletion improved survival and reduced T cell IFNγ production, suggesting FXR can transcriptionally promote T cell effector functions in certain inflammatory settings (88).

FXR’s role as a master transcriptional regulator of metabolic genes is also critical in the TIME. Canonically, it controls BA homeostasis by regulating genes involved in BA synthesis (CYP7A1, CYP8B1), transport (BSEP, ASBT, OSTα/β), and feedback regulation (SHP, FGF15/19) (84, 85, 90). Dysregulation of this metabolic programming is implicated in liver diseases and cancer, with the FXR-FGF19 axis often altered in NASH-associated HCC (91). Furthermore, FXR transcriptionally activates Acsl4 in intestinal epithelial cells (IECs), promoting ferroptosis and subsequently impairing ILC3 function, and can induce Wnt2b in cancer-associated fibroblasts (CAFs) within the tumor stroma, fostering a pro-tumorigenic niche (92, 93).

Crucially, FXR signaling is often dysregulated in intestinal inflammation and cancer. Reduced FXR expression or altered activity contributes to the pathogenesis of IBD, CRC, and GEAC (28, 89, 90), positioning FXR as a potential tumor suppressor in the gastrointestinal tract (28, 90).

In summary, FXR integrates microbial and metabolic signals (primarily BAs) to exert transcriptional control over immune and metabolic genes across various cell types within the TIME. Its activity, modulated by ligands and upstream immune pathways, dictates transcriptional outcomes influencing intestinal homeostasis, inflammation, metabolism, and carcinogenesis.

#### 2.3.2 Takeda G protein-coupled Receptor 5: a complex regulator of inflammatory responses and cell function

Distinct from the nuclear receptor FXR discussed previously, Takeda G protein-coupled Receptor 5 (TGR5, or GPBAR1) serves as a primary cell surface sensor for secondary bile acids (SBAs), particularly lithocholic acid (LCA) and deoxycholic acid (DCA), which are generated through gut microbial metabolism (2, 77). TGR5 is expressed by various immune cells crucial within the TIME, including macrophages and dendritic cells (DCs), as well as by certain cancer cells and intestinal epithelial cells, but notably not T lymphocytes (2, 94, 95). This distribution allows TGR5 to mediate crosstalk between microbial metabolites and host immunity.

Activation of TGR5 classically triggers Gαs-cAMP-PKA signaling, which often exerts anti-inflammatory effects. This pathway typically suppresses NF-κB activation and NLRP3 inflammasome activity, leading to reduced production of key pro-inflammatory cytokines (e.g., TNF-α, IL-6, IL-12) by macrophages and DCs (2, 77, 96). Consequently, TGR5 signaling promotes M2 macrophage polarization, fosters tolerogenic DC phenotypes, and contributes to intestinal barrier integrity and epithelial regeneration (2, 97, 98). These anti-inflammatory actions are implicated in the protective roles observed for TGR5 activation or SBA modulation in models of colitis (97, 99), rheumatoid arthritis (79), and diabetic retinopathy (100).

However, the net effect of TGR5 activation within the TIME is complex and highly context-dependent. TGR5 can exhibit biased agonism, preferentially activating either Gαs or alternative pathways (e.g., β-arrestin) depending on the specific ligand (95). This signaling flexibility, known as biased agonism, is increasingly recognized as a key factor underlying its potentially dual role in cancer. For instance, while LCA activation of TGR5 induced cytostatic oxidative stress in breast cancer cells (94), SBA activation of TGR5 on colorectal cancer cells promoted Treg recruitment via β-catenin/CCL28 signaling, facilitating immune evasion (74). Furthermore, β-arrestin-biased TGR5 signaling was shown to promote NSCLC cell proliferation via YAP activation, contrasting with the anti-proliferative effects of Gs-biased signaling (95). In other contexts, like a specific NASH model, TGR5 activation appeared less critical than FXR activation for therapeutic benefit (85), and in a colon cancer metastasis model, TGR5 downregulation correlated with improved outcomes (101). TGR5 expression has also been linked to gastric cancer severity in humans, though its functional role requires further study (102).

In conclusion, TGR5/GPBAR1 is a pivotal receptor linking microbiota-derived SBAs to the modulation of inflammation and cell function within the TIME. Its canonical anti-inflammatory pathway contrasts with context-specific or biased signaling mechanisms that can contribute to either tumor suppression or promotion, highlighting the critical need to understand these nuances when considering TGR5 as a therapeutic target.

#### 2.3.3 Sphingosine-1-phosphate receptor 2: potential crosstalk with conjugated bile acids in TIME

Beyond receptors primarily sensing unconjugated or secondary BAs, the G protein-coupled receptor Sphingosine-1-Phosphate Receptor 2 (S1PR2) represents another signaling node potentially linking BA metabolism to the TIME, primarily through interactions with conjugated bile acids (CBAs) (2, 29). Unlike FXR and TGR5, S1PR2’s primary ligand is sphingosine-1-phosphate (S1P), but studies suggest that CBAs, such as taurocholic acid (TCA) and taurochenodeoxycholic acid (TCDCA), may act as modulators or activators of S1PR2, particularly in pathological settings (2, 29, 103). S1PR2 is expressed on various cell types relevant to the TIME, including myeloid cells (macrophages, monocytes, neutrophils) and certain cancer cells, positioning it to potentially influence tumor-associated inflammation and progression (2, 29, 103).

While S1PR2 is known for its complex roles in regulating immune cell trafficking (often antagonizing S1PR1) (2, 29), direct evidence linking CBA-mediated S1PR2 activation to specific immune cell functions within the TIME is still emerging and less characterized compared to FXR and TGR5 (2). However, compelling evidence connects the CBA-S1PR2 axis to cancer cell behavior and inflammation in relevant contexts. Notably, in esophageal adenocarcinoma (EAC) cells, TCA was shown to promote invasive growth, epithelial-mesenchymal transition (EMT), and cancer stem cell expansion specifically through S1PR2 activation, a process involving downstream YAP and β-catenin signaling pathways (103). Furthermore, in patients with HCV-related chronic liver disease, levels of taurine-conjugated BAs strongly correlated with increased hepatic S1PR2 expression, markers of inflammation (NLRP3 inflammasome, NF-κB pathway genes), and liver disease severity, suggesting a pathological role for the Tau-BA-S1PR2 axis in hepatic inflammation (104). These findings highlight a potential pro-tumorigenic and pro-inflammatory role for S1PR2 when activated, possibly by elevated CBAs in specific microenvironments.

The role of S1PR2 appears highly context-dependent, potentially exerting opposing effects in different tissues or disease states (29). Compared to the more established roles of FXR and TGR5, the precise contribution of the BA-S1PR2 interaction to shaping the overall immune landscape within the TIME warrants further investigation (2).

In summary, S1PR2 adds another layer of complexity to BA signaling within the TIME, potentially acting as a receptor for conjugated BAs to influence cancer cell invasiveness and inflammation, possibly contributing to a pro-tumorigenic microenvironment in certain settings.

#### 2.3.4 Vitamin D receptor: interaction with bile acid signaling in immunity

The Vitamin D Receptor (VDR, NR1I1) adds another layer of complexity to the bile acid (BA) signaling network within the immune system. Beyond its canonical role in mediating vitamin D actions, VDR functions as a direct sensor for specific gut microbiota-derived secondary BAs, notably lithocholic acid (LCA) and its oxidized derivative 3-oxoLCA (71, 105). This positions VDR to integrate signals from both endocrine vitamin D and microbial BA metabolism.

A key aspect of VDR’s interaction with BA signaling involves the direct modulation of adaptive immunity. LCA and 3-oxoLCA, acting through VDR expressed intrinsically on T cells, are crucial for maintaining the homeostasis of colonic RORγt+ regulatory T cells (Tregs), a population vital for intestinal immune tolerance and controlling colitis (71). LCA has also been reported to inhibit Th1 differentiation via VDR signaling (77). Furthermore, VDR signaling is integral to maintaining intestinal barrier integrity by regulating key tight junction proteins (106). This barrier function is fundamental for shaping the local immune microenvironment and preventing aberrant immune activation, a role potentially influenced by BA ligands. Importantly, the immunomodulatory effects of LCA metabolites display receptor specificity; for instance, isoalloLCA enhances Treg differentiation via VDR-independent pathways involving mitochondrial ROS or NR4A1 (70, 77), while LCA can exert cytostatic effects in cancer cells through TGR5 and CAR (Constitutive Androstane Receptor, NR1I3) (94). This highlights that VDR mediates distinct, rather than universal, effects of LCA metabolites on immune cells.

Furthermore, VDR signaling pathways exhibit direct molecular crosstalk and functional competition with other key nuclear receptors sensing BAs and xenobiotics. Seminal studies revealed that VDR, Farnesoid X Receptor (FXR), and Pregnane X Receptor (PXR) can all bind to the same cis-regulatory element, an imperfect inverted repeat (IR0), within the promoter of the Sult2A1 gene, which encodes a sulfotransferase involved in LCA detoxification (107, 108). This convergence on a shared DNA binding site leads to functional antagonism, where FXR or PXR activation can competitively inhibit VDR-mediated transcription from this element (107). VDR signaling might also indirectly influence BA signaling by transcriptionally upregulating the BA-metabolizing enzyme CYP3A4 and efflux transporters like MDR1 and MRP2 (109).

In essence, VDR participates actively in the BA-immune axis, acting as a specific receptor for LCA/3-oxoLCA to regulate key adaptive immune cells like RORγt+ Tregs. Crucially, it engages in direct molecular interactions and competition with FXR and PXR at shared gene regulatory elements, providing a clear mechanism for crosstalk between these major BA-sensing pathways. These interactions underscore VDR’s role as an integral node in the complex network governing BA signaling and its impact on immunity, particularly within the intestinal microenvironment relevant to inflammation and cancer (105, 108, 110).

### 2.4 The gut microbiota: orchestrating the bile acid pool and shaping the TIME

The intestinal microbiota functions as a sophisticated metabolic organ that profoundly influences the TIME. While its capacity to reshape the host’s bile acid (BA) landscape is a central theme of this review, it is important to recognize that bacteria also employ direct, non-metabolite-driven mechanisms to modulate tumorigenesis. A prime example of a pro-tumorigenic bacterium is *Fusobacterium nucleatum*, a commensal of the oral cavity that is frequently enriched in colorectal cancer (CRC) tissues. Its translocation to the gut allows it to promote tumorigenesis through multiple mechanisms, including the engagement of its FadA adhesin with host E-cadherin, which activates Wnt/β-catenin signaling and fosters an inflammatory microenvironment (111). This example highlights the concept of microbial translocation from other niches, such as the oral cavity, influencing gastrointestinal tumorigenesis, a phenomenon also observed for other pathogens like *Porphyromonas gingivalis* in pancreatic cancer (112).

Alongside such direct interactions, the microbial “orchestration” of the BA landscape—through a cascade of enzymatic modifications including deconjugation by bile salt hydrolases (BSH) (5), 7α-dehydroxylation by bai operon-encoded enzymes (113, 114), various epimerizations and oxidations by hydroxysteroid dehydrogenases (HSDHs) (115), as well as 5α-reduction leading to allo-bile acids (Allo-BAs) (116)—generates a diverse pool of secondary and modified BAs whose altered structures confer distinct signaling capacities, enabling them to interact with host receptors and profoundly influence the TIME (2, 16). The specific microbial consortia and their BA-modifying activities are themselves dynamically regulated by host factors (44), diet (74, 100), and medications (38, 117), highlighting a complex interplay.

Microbially-derived BAs can significantly sculpt the TIME by modulating key immune cell populations. For instance, deoxycholic acid (DCA) and lithocholic acid (LCA), produced by 7α-dehydroxylating bacteria like *Clostridium scindens (* 67, 74), often promote an immunosuppressive TIME. DCA directly impairs CD8+ T cell effector functions by enhancing PMCA activity and subsequently inhibiting Ca2+-NFAT2 signaling (67). Both DCA and LCA can also trigger tumor cell TGR5 activation, leading to CCL28-mediated recruitment of regulatory T cells (Tregs) (74, 118). Specific microbial shifts, such as an increase in *Bacteroidetes ovatus* following host AKR1D1 loss, can lead to the accumulation of isolithocholic acid (iso-LCA), which suppresses NK cell cytotoxicity and survival, potentially via TGR5 (44). Conversely, isodeoxycholic acid (isoDCA), generated through complex microbial biotransformations involving bacteria such as Clostridium scindens, Eggerthella lenta, and Ruminococcus gnavus (54, 55), can act on dendritic cells (DCs), possibly by antagonizing FXR, to promote the differentiation of RORγt+ pTregs (55). The newly identified allo-BAs, produced by Firmicutes harboring BaiP/J genes (119), are also enriched in colorectal cancer (CRC) and their derivatives, such as isoalloLCA (70) and 3-oxolithocholic acid (3-oxoLCA) (71), show potent immunomodulatory activities, primarily by influencing the Treg/Th17 balance, thereby significantly shaping the TIME.

However, the microbiota-BA axis can also generate signals conducive to anti-tumor immunity or complex immune regulation. A relative increase in primary BAs (e.g., CA, CDCA) over secondary BAs, often resulting from alterations in 7α-dehydroxylating bacteria (e.g., due to antibiotics or traditional medicines like XYXD), can enhance anti-tumor NKT cell responses by promoting CXCL16 expression on liver sinusoidal endothelial cells (LSECs) and subsequent CXCR6+ NKT cell recruitment and IFN-γ production (120, 121). Furthermore, certain BAs like ursodeoxycholic acid (UDCA) and ursocholic acid (UCA), associated with *Lachnoclostridium* enrichment, correlate with improved outcomes in HCC patients receiving immunotherapy (119). UDCA and its potential microbial metabolite tauroursodeoxycholic acid (TUDCA) can modulate host inflammation through TGR5 activation (and in some contexts, FXR activation) leading to downstream effects such as reduced TNF-α and modulation of NF-κB signaling (96, 100). Additionally, conjugated BAs (e.g., TCA, TCDCA), whose levels are influenced by microbial BSH activity, can directly suppress macrophage IL-6 production, a key cytokine in the TIME (38).

The gut microbiota itself adapts to the BA environment it helps create. High concentrations of cytotoxic BAs (e.g., DCA) can drive microbial transcriptional reprogramming, including upregulation of efflux pumps and stress response genes, and alterations in core metabolism and BA-modifying enzyme expression (122). These adaptations, along with the co-production of other immunomodulatory metabolites like short-chain fatty acids (SCFAs), create a complex metabolic network. Butyrate, a key SCFA, exemplifies this complexity through the ‘butyrate paradox’: in normal colonocytes, it serves as a primary energy source and acts as a histone deacetylase (HDAC) inhibitor, thereby promoting gut health (123). In the context of CRC, however, its role is multifaceted. While some cancer cells can utilize it as fuel, butyrate has also been shown to counteract the pro-tumorigenic effects of deoxycholic acid (DCA) by specifically inhibiting the proliferation of DCA-resistant cancer cells (124). The functional heterogeneity within key BA-metabolizing families like Lachnospiraceae further underscores the complexity of this network (125).

In essence, the gut microbiota, through its sophisticated enzymatic machinery and dynamic interplay with the host, orchestrates the BA pool, transforming BAs into a diverse array of signaling molecules. These microbiota-derived BA signals, often in concert with other microbial metabolites, engage multiple host targets to profoundly shape the cellular composition, functional orientation, and therapeutic susceptibility of the TIME, offering novel avenues for cancer diagnosis and intervention (118, 119, 126).

The preceding sections (2.1–2.4) have systematically delineated how the gut microbiota, through its ability to convert host-derived primary bile acids into a diverse spectrum of secondary and modified BAs, serves as a critical upstream regulator of the TIME. These microbiota-shaped BAs, by engaging a repertoire of host receptors—including FXR, TGR5, VDR, and S1PR2—on immune, stromal, and cancer cells, collectively orchestrate a complex signaling network. This network dynamically modulates the differentiation, activation, and effector functions of key innate and adaptive immune populations, ultimately shaping the tumo’s immunological landscape. Frequent dysregulation of this intricate microbiota–BA–immune axis in cancer often results in an immunosuppressive TIME that facilitates tumor progression and immune evasion. Recognizing the pathological impact of these multifaceted interactions provides a compelling rationale for therapeutically targeting this axis to reprogram anti-tumor immunity—a strategy explored in the next section.

Before delving into these therapeutic strategies, **Table 1** summarizes the bile acid-mediated regulatory mechanisms discussed above, highlighting their effects on key immune cell populations within the TIME and their immunological implications in cancer.

**Table 1:** Immunomodulatory effects of bile acids on innate and adaptive immune cells in the tumor immune microenvironment (TIME).

Bile acid (BA)	Source/classification	Target immune cell(s)	Key receptor(s) involved	Key immunomodulatory effects	Implications in cancer immunity
Chenodeoxycholic acid (CDCA)	Primary (host-synthesized)	Macrophages	FXR	↓ Pro-inflammatory cytokine secretion (e.g., TNF-α, IL-1β, IL-6; via NF-κB/NLRP3 inhibition); ↑ M2 polarization.	Anti-inflammatory; cancer implications are context-dependent.
Ursodeoxycholic acid (UDCA)	Secondary (microbial-derived)/Therapeutic	Macrophages, Dendritic Cells (DCs)	FXR (context-dependent modulation) , TGR5 (potential)	Macrophages: ↑ M2 polarization; ↓ Pro-inflammatory cytokine secretion. DCs: ↑ Antigen presentation capacity (*in vitro*, with ILDR).	Therapeutic potential: improves ICI outcomes (HCC), enhances DC antigen presentation (with ILDR); context-dependent FXR/TGR5 modulation.
Lithocholic acid (LCA)	Secondary (microbial-derived)	Macrophages, DCs, Tregs, Th1 cells, Th17 cells	TGR5 (Macrophages, DCs); VDR (Tregs, Th1 cells); RORγt (Th17 cells, via derivatives e.g., 3-oxoLCA)	Macrophages/DCs (TGR5): ↓ Pro-inflammatory cytokine secretion (e.g., TNF-α, IL-12, IL-6); ↑ M2 polarization; ↑ IL-10 production. Tregs (VDR, with 3-oxoLCA): Maintains colonic RORγt+ Treg homeostasis. Th1 cells (VDR): ↓ Differentiation. Th17 cells (RORγt antagonist): ↓ Differentiation & IL-17 production.	Context-dependent; Can promote immunosuppressive TIME in CRC (e.g., via tumor cell TGR5 activation → ↑ Treg recruitment, associated with poor outcomes).
Deoxycholic acid (DCA)	Secondary (microbial-derived)	Macrophages, DCs, MDSCs, CD8^+^ T cells, Plasmacytoid DCs (pDCs)	TGR5 (Macrophages, DCs, Tumor cells); PMCA (CD8^+^ T cells); TLR7 (pDCs); FXR (MDSCs, potential)	Macrophages/DCs (TGR5): ↓ Pro-inflammatory cytokine secretion; ↑ M2 polarization. Macrophages (high conc.): ↑ M2-like TAM polarization. MDSCs: ↑ Recruitment & activation (with LPS). CD8^+^ T cells (PMCA): ↓ Cytotoxicity (↓ IFN-γ, TNF-α, GzmB). pDCs (TLR7): ↑ IFN-I production.	Largely pro-tumorigenic: promotes M2 TAMs, MDSC expansion, and Treg recruitment (CRC); impairs CD8^+^ T cell cytotoxicity; implicated in CRC liver metastasis.
Isolithocholic acid (iso-LCA)	Secondary (microbial-derived)	NK cells	TGR5 (putative; validation pending for NK cell effects)	↓ NK cell cytotoxicity (↓ IFN-γ, TNF-α; via p-CREB1 inhibition); ↑ NK cell apoptosis.	Pro-tumorigenic via suppression of NK cytotoxicity and survival; antagonism may enhance ICI efficacy (HCC).
Taurocholic acid (TCA)	Conjugated (Taurine; primarily host-synthesized, modulated by microbiota)	MDSCs, Macrophages, Cancer cells (context-dependent)	FXR (MDSCs, potential), S1PR2 (Cancer cells, specific contexts)	MDSCs (FXR)?: ↑ M-MDSC expansion & immunosuppressive function (↑ glycolysis, PD-L1 stabilization). Macrophages: ↓ IL-6 production. Cancer cells (S1PR2, EAC): ↑ Invasion, EMT, CSC expansion.	Promotes MDSC-mediated immunosuppression in CRC lung metastasis. Pro-tumorigenic in EAC via S1PR2.
Isodeoxycholic acid (isoDCA)	Secondary (microbial-derived)	Dendritic Cells (DCs)	FXR (potential antagonist or biased agonist on DCs)	↓ Antigen presentation machinery (e.g., MHC-II); ↓ Pro-inflammatory cytokine secretion (e.g., TNF-α, IL-6); ↑ Peripheral Treg differentiation (indirectly via DCs).	Potentially immunosuppressive by promoting tolerogenic DCs and pTreg differentiation.
3-Oxolithocholic acid (3-oxoLCA)	Secondary (microbial-derived)	Tregs, Th17 cells	VDR (Tregs, in combination with LCA); RORγt (Th17 cells, as antagonist)	Tregs (VDR): Maintains colonic RORγt+ Treg homeostasis. Th17 cells (RORγt antagonist): ↓ Differentiation.	Promotes Treg/Th17 balance and mucosal immune tolerance; generally anti-inflammatory, though cancer-specific roles remain under investigation.
IsoalloLCA	Secondary (microbial-derived)	Tregs, Th17 cells	VDR-independent; mitoROS/CNS3-dependent pathway (Tregs); RORγt-independent mechanism (Th17 cells)	Tregs: ↑ Peripheral Treg differentiation (enhances FoxP3 expression; VDR-independent). Th17 cells: Exhibits some *in vitro* inhibition of differentiation.	Primarily promotes Treg differentiation (fostering immune tolerance); Role in cancer TIME under investigation.
Taurolithocholic acid (TLCA)	Secondary (microbial-derived)/Conjugated (Taurine)	T cells (including CD8^+^ T cells)	(Receptor mediating direct T cell effects not fully elucidated)	↑ T cell activation, proliferation, and effector cytokine expression.	Associated with improved ICI efficacy (NSCLC), potentially via memory CD8^+^ T cell promotion; Potential as an ICI adjuvant.
Tryptophan-conjugated CDCA (e.g., Trp-CDCA)	Modified (microbial amino acid-conjugated)	CD4^+^ T cells	(Receptor unknown)	↑ IFN-γ production *in vitro* (Th1-skewing).	Potential Th1-polarizing and anti-tumor role; further investigation needed in cancer models.

This table summarizes the immunomodulatory effects of representative bile acids (BAs), including primary, secondary, conjugated, and microbiota-modified form, on major innate and adaptive immune cell populations within the tumor immune microenvironment (TIME). For each BA, its origin, immune cell targets, receptor involvement, major regulatory functions, and cancer-related immunological implications are outlined. Mechanistic insights and functional annotations are based on findings discussed in Section 2 of the main text. Detailed citations can be found in Section 2.

3-oxoLCA, 3-oxolithocholic acid; BA, bile acid; BSH, bile salt hydrolase; CA, cholic acid; CBA, conjugated bile acid; CDCA, chenodeoxycholic acid; CRC, colorectal cancer; CSC, cancer stem cell; DCA, deoxycholic acid; DC, dendritic cell; EMT, epithelial–mesenchymal transition; FXR, farnesoid X receptor; GzmB, granzyme B; IFN, interferon; isoDCA, isodeoxycholic acid; isoLCA, isolithocholic acid; LCA, lithocholic acid; MDSC, myeloid-derived suppressor cell; NK, natural killer (cell); PBA, primary bile acid; PMCA, plasma membrane Ca²^+^-ATPase; RORγt, RAR-related orphan receptor gamma t; S1PR2, sphingosine-1-phosphate receptor 2; SBA, secondary bile acid; TAM, tumor-associated macrophage; TCA, taurocholic acid; TGR5, Takeda G-protein-coupled receptor 5; TLCA, taurolithocholic acid; TNF, tumor necrosis factor; Treg, regulatory T cell; Trp-CDCA, tryptophan-conjugated CDCA; UDCA, ursodeoxycholic acid; VDR, vitamin D receptor.

## 3 Therapeutic targeting of the microbiota-bile acid-immune axis

**Figure 3:**
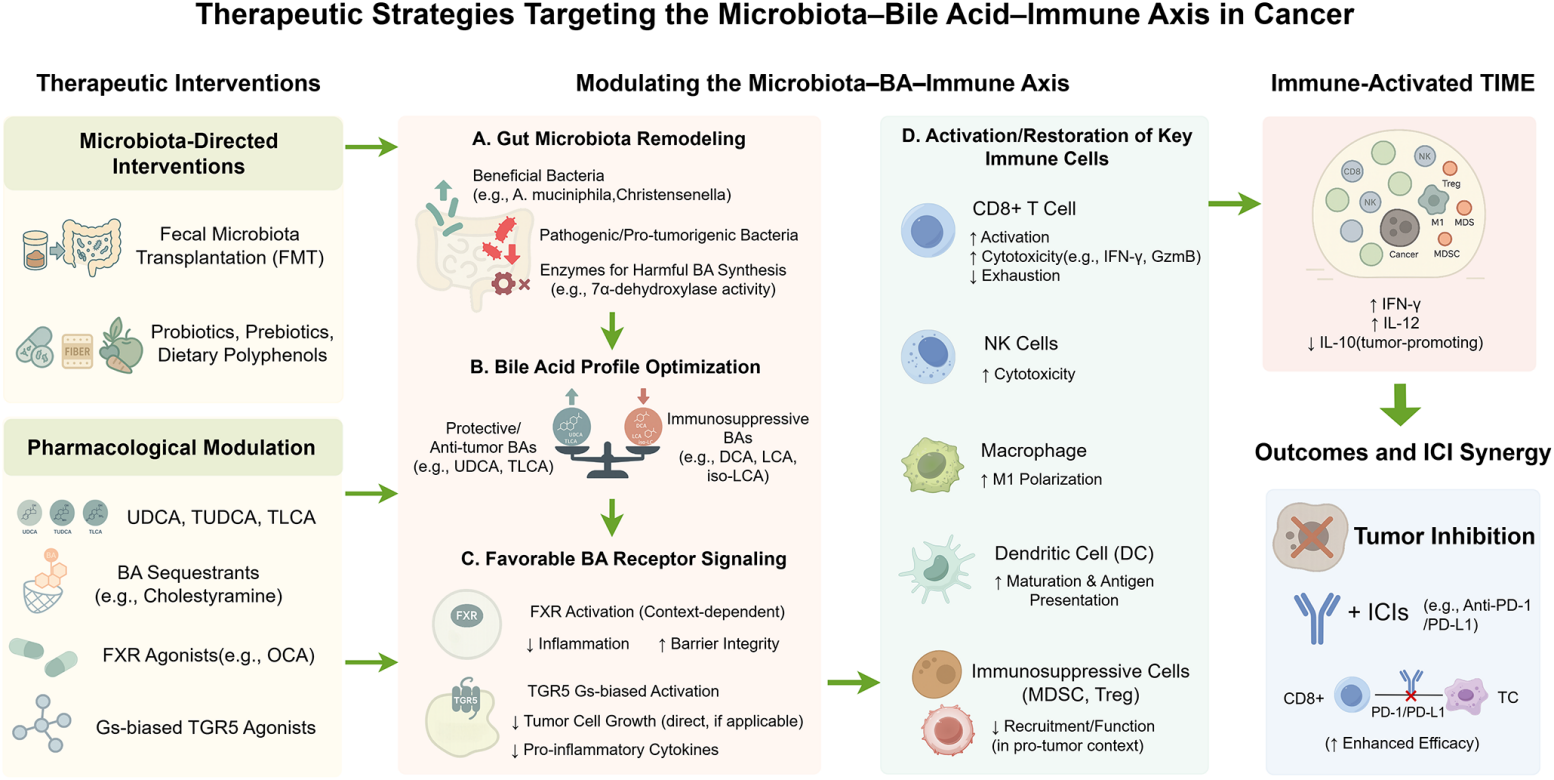
Therapeutic strategies targeting the microbiota–bile acid–immune axis in cancer. This schematic summarizes therapeutic strategies designed to modulate the microbiota–bile acid (BA)–immune axis to reprogram the tumor immune microenvironment (TIME) and enhance anti-tumor immunity. The figure presents a left-to-right flow: interventions [e.g., probiotics, polyphenols, fecal microbiota transplantation (FMT), Farnesoid X receptor (FXR)/Takeda G-protein-coupled receptor 5 (TGR5) modulators, bile acid sequestrants], microbiota and BA metabolism, and downstream immune effects. These interventions alter microbial composition (e.g., ↑ A. muciniphila) and modulate BA-metabolizing enzyme activity [e.g., bile salt hydrolase (BSH), 7α-dehydroxylase], resulting in a favorable BA profile—↑ protective BAs [e.g., ursodeoxycholic acid (UDCA), taurolithocholic acid (TLCA)], ↓ immunosuppressive BAs [e.g., deoxycholic acid (DCA), lithocholic acid (LCA), isolithocholic acid (iso-LCA)]—and context-specific receptor signaling (e.g., FXR, TGR5). Immune cell function is reprogrammed: CD8^+^ T cells regain activation and cytotoxicity [e.g., ↑ interferon-γ (IFN-γ), granzyme B], natural killer (NK) cells recover function, macrophages polarize toward M1, dendritic cells (DCs) enhance antigen presentation, and suppressive populations [e.g., regulatory T cells (Tregs), myeloid-derived suppressor cells (MDSCs)] are reduced. These shifts remodel the TIME into an immune-activated state and synergize with immune checkpoint inhibitors [ICIs; e.g., anti-Programmed Death-1 (PD-1)/Programmed Death-Ligand 1 (PD-L1)], potentially overcoming therapeutic resistance. Color-coded arrows indicate functional modulation: green for activation or enhancement; red for suppression or inhibition. By Figdraw.

**Figure 3** outlines therapeutic strategies targeting the microbiota–bile acid–immune axis and their mechanisms for enhancing anti-tumor immunity.

### 3.1 Pharmacological modulation of bile acid signaling

Pharmacological targeting of bile acid (BA) signaling, a critical regulator of the TIME (2, 4), offers promising therapeutic avenues for cancer. Strategies target key BA-activated receptors (BARRs) like Farnesoid X Receptor (FXR) and Takeda G protein-coupled Receptor 5 (TGR5/GPBAR1), utilize specific BAs with immunomodulatory actions, or indirectly alter the BA pool via sequestrants, all with the potential to reshape the TIME.

#### 3.1.1 Farnesoid X receptor modulators: a context-dependent balancing act

FXR, pivotal in BA homeostasis and inflammation, presents a dual-faceted therapeutic target (84, 127). FXR agonists, like obeticholic acid (OCA), have shown preclinical efficacy in gastroesophageal adenocarcinoma (GEAC). OCA treatment ameliorated high-fat diet (HFD)-induced dysplasia, potentially by reducing deleterious secondary BAs (e.g., DCA, TDCA), decreasing microbial bile salt hydrolase (BSH) expression, and favorably reshaping the tumor’s immune landscape (90, 128), thereby potentially reversing an immunosuppressive TIME. OCA also mitigates liver fibrosis (84, 129), though clinical use faces challenges from side effects like pruritus (130, 131). Moreover, a key challenge is balancing the desired anti-tumor immune effects with potential systemic metabolic side effects, such as dyslipidemia, which has been observed with some FXR agonists (132). Other agonists, such as the intestine-specific Fexaramine D (FexD), suppressed colitis-associated cancer (CAC) by restoring FXR signaling, enhancing intestinal barrier integrity, and reducing pro-inflammatory M1-like macrophage infiltration within the TIME (81).

Conversely, FXR inhibition or functional antagonism demonstrates therapeutic value where FXR activation is detrimental. In radiation-associated hematopoietic recovery (RAHR), *Bacteroides* acidifaciens-driven BA deconjugation activated FXR, impairing NF-κB-dependent recovery; this was rescued by ursodeoxycholic acid (UDCA), acting as an FXR inhibitor in this context (133). Similarly, in T cell-driven graft-versus-host disease (GVHD), FXR agonism exacerbated mortality, while T cell-specific FXR deletion or UDCA administration (again exhibiting FXR antagonistic properties) improved outcomes (88). The therapeutic strategy for FXR modulation (activation vs. inhibition) is thus highly context-dependent, influenced by the specific pathology and its interplay with the TIME.

#### 3.1.2 Takeda G protein-coupled receptor 5 modulators: harnessing biased agonism

TGR5, predominantly activated by secondary BAs like lithocholic acid (LCA) and DCA, exerts immunomodulatory effects (2, 77) often dictated by biased agonism. This phenomenon, where ligands preferentially activate Gαs-cAMP or β-arrestin pathways, leads to divergent biological outcomes crucial in cancer pharmacology (95). For instance, in non-small cell lung cancer (NSCLC), Gαs-biased TGR5 activation by INT-777 or DCA inhibited YAP activity and suppressed proliferation. In contrast, the β-arrestin 1-biased agonist R399 activated YAP, promoting NSCLC growth (95), suggesting a therapeutic window for Gs-biased TGR5 agonists. In rheumatoid arthritis models, probiotic-induced DCA/LCA activated TGR5 (likely Gs-cAMP mediated given the anti-inflammatory outcome) to suppress Th1/Th17 responses, an effect nullified by the TGR5 antagonist SBI-115 (79). Therefore, designing TGR5 ligands with specific signaling bias is paramount for achieving desired anti-inflammatory or anti-tumor efficacy within the TIME.

#### 3.1.3 Therapeutic utility of specific bile acids: UDCA/TUDCA and emerging candidates

Beyond their roles as ligands for specific BARRs, certain BAs themselves are used or being investigated as therapeutic agents due to their pleiotropic effects. The pharmacological actions of UDCA are notably complex and appear highly dependent on the specific cellular and disease context, potentially involving differential engagement of FXR as either an agonist or antagonist, alongside other mechanisms. Ursodeoxycholic acid (UDCA) and its taurine conjugate, tauroursodeoxycholic acid (TUDCA), are well-established for their cytoprotective and anti-inflammatory properties (96, 97). As discussed, UDCA’s effects on FXR appear context-dependent, exhibiting inhibitory functions in RAHR and GVHD (88, 133), while potentially mediating anti-inflammatory effects in colonic inflammation through FXR activation and M2 macrophage polarization (32). TUDCA mitigated aGVHD by reducing intestinal epithelial apoptosis and downregulating antigen presentation by non-hematopoietic cells, independently of microbiome changes and without compromising graft-versus-leukemia effects (61). The multifaceted actions of UDCA/TUDCA (e.g., contextual FXR modulation, TGR5 agonism (79, 100), direct cytoprotection) underscore their therapeutic versatility in modulating immune responses.

Emerging research highlights other BAs. In NSCLC patients on ICIs, elevated plasma taurolithocholic acid (TLCA) and glycochenodeoxycholic acid (GCDCA) correlated with improved outcomes. Notably, TLCA enhanced T cell activation and anti-tumor immunity, suggesting its potential to favorably modulate the TIME and act as an ICI adjuvant (68). The gut microbe *Eubacterium* spp. was linked to TLCA levels (68), pointing to microbial influence on these immunomodulatory BAs.

#### 3.1.4 Bile acid sequestrants: indirectly modulating BA signaling

Bile acid sequestrants (BASs) like cholestyramine offer an indirect strategy to modulate BA signaling. By binding intestinal BAs and promoting their excretion, BASs reduce the BA pool returning to the liver, potentially decreasing BA-induced hepatotoxicity and altering systemic BA signals (134). In a model of dysbiosis and high soluble fiber intake leading to cholestatic liver cancer, cholestyramine prevented HCC development (134). By modifying ligand availability for BARRs, BASs may have utility in BA-driven pathologies, though their specific impact on the TIME warrants further investigation.

In summary, pharmacological strategies targeting BA signaling offer a diverse toolkit to reshape the TIME and influence cancer immunity. Successful clinical translation, however, requires a nuanced understanding of the context-dependent actions of BAs, specific BA species, receptor expression, signaling bias, and the intricate BA-microbiota-immune axis within the disease microenvironment, paving the way for novel immunotherapeutic interventions.

### 3.2 Microbiota-based strategies to modulate bile acids and shape anti-tumor immunity

The profound capacity of the gut microbiota to sculpt the bile acid (BA) pool (Section 2.4) offers a rich landscape for therapeutic interventions aimed at reshaping the TIME. By strategically modulating the microbiota, it is possible to alter the intricate balance of primary and secondary BAs, their conjugation status, and the abundance of specific immunomodulatory species. These alterations, in turn, can engage host BA receptors like Farnesoid X Receptor (FXR) and Takeda G protein-coupled Receptor 5 (TGR5/GPBAR1) on immune and cancer cells, thereby influencing anti-tumor immune responses and the efficacy of treatments such as immune checkpoint inhibitors (ICIs). This section explores key microbiota-targeted approaches, from engineered bacteria to dietary interventions, focusing on their potential to therapeutically harness the microbiota-BA-immune axis in oncology, a field actively exploring microbial strategies to enhance cancer immunotherapy (14).

#### 3.2.1 Engineered microbes and microbial enzymes for precision BA immunomodulation

Synthetic biology enables the engineering of bacteria or the targeting of specific microbial enzymes for precise BA modulation and subsequent immune programming. For instance, a potential strategy involves engineering bacteria to enhance the production of beneficial BAs like isodeoxycholic acid (isoDCA). The rationale for such an approach is supported by studies such as Campbell C et al. (2020), who demonstrated that naturally microbially-derived isoDCA promotes peripheral regulatory T cell (pTreg) generation by acting on dendritic cells (DCs) as a functional antagonist of FXR, thereby inducing a tolerogenic DC phenotype (55). Conversely, inhibiting detrimental microbial BA metabolism is also a viable strategy. Sun L et al. (2023) showed that bile salt hydrolase (BSH) activity in non-enterotoxigenic *Bacteroides* potentiated colorectal cancer (CRC) by increasing colonic deoxycholic acid (DCA) and lithocholic acid (LCA), which activated tumor cell TGR5, upregulated CCL28, and promoted immunosuppressive Treg infiltration. Pharmacological BSH inhibition reversed these pro-tumorigenic effects (74). Furthermore, specific microbial activities can have broader systemic impacts; Jiao Y et al. (2025) found that *Bacteroides acidifaciens*-driven BA deconjugation activated host FXR, impairing NF-κB-dependent hematopoietic recovery post-radiation, an effect rescued by the FXR inhibitor ursodeoxycholic acid (UDCA) (133). Given the importance of hematopoietic integrity for sustained anti-tumor immunity, especially post-cytotoxic therapies, such microbial BA modulations impacting host recovery warrant attention in oncological contexts (133). These studies collectively underscore that precise manipulation of microbial BA metabolic pathways, whether by enhancing beneficial BA production or inhibiting detrimental BA formation, can significantly impact host immune homeostasis and disease progression. However, developing engineered microbes as live biotherapeutic products presents considerable challenges in regulatory affairs, manufacturing, genetic stability, biocontainment, delivery, engraftment, and overall safety (135).

#### 3.2.2 Fecal microbiota transplantation for restoring BA-mediated immune competence

Fecal Microbiota Transplantation (FMT) aims to restore a healthy microbial ecosystem and its BA biotransformation capabilities. Buffie CG et al. (2015) classically demonstrated that FMT, or even reconstitution with keystone species like *Clostridium scindens*, restores resistance to CDI by increasing secondary BAs like DCA and LCA (114). The functionality of such BA-transforming bacteria is dynamically regulated; for instance, the bai operon in C. scindens, crucial for DCA generation, is induced by primary BAs but not DCA itself (136). In oncology, FMT has shown promise in improving ICI efficacy, with studies in melanoma (137, 138) and other cancers (14) linking positive responses to favorable microbiota shifts and potentially altered BA metabolism. However, the outcomes of FMT can be complex and context-dependent. Rashidi A et al. (2024) reported an unexpected higher incidence of acute graft-versus-host disease (aGVHD) post-FMT in alloHCT recipients, associated with *Faecalibacterium* expansion and an inverse correlation with the anti-inflammatory BA, UDCA (139). This highlights the critical need for a nuanced understanding of FMT’s impact on the microbiota-BA-immune axis and the potential necessity for more precision-engineered approaches to enhance predictability and targeting (140–142).

#### 3.2.3 Dietary interventions, prebiotics, and probiotics in shaping the BA-immune landscape

Dietary interventions are fundamental for modulating the gut microbiota and BA pool. O’Keefe SJ et al. (2015) demonstrated that a 2-week dietary swap between African Americans and rural Africans led to rapid, reciprocal changes in fecal secondary BA levels (DCA, LCA), butyrogenesis, and colonic inflammation/proliferation markers, underscoring diet’s potent impact on BA metabolism and cancer risk indicators (143). However, dietary effects are context-dependent; high soluble fiber intake in dysbiotic mice induced cholestatic HCC via dysregulated microbial fermentation and altered BA homeostasis (134), while a high-fat, high-cholesterol diet post-cholecystectomy exacerbated BA dysregulation and intestinal inflammation (144).

Targeted prebiotics and probiotics offer more specific modulation. Natural polyphenols like castalagin (145) and cranberry proanthocyanidins (146) act as prebiotics, reshaping gut microbiota to alter BA profiles (e.g., increasing taurine-conjugated BAs or reducing local BA accumulation, respectively) and exert anti-tumor effects or enhance ICI responsiveness. Inulin, particularly in combination with rifaximin, suppressed colon cancer metastasis by fostering beneficial BA-related bacteria (*Eubacterium*), reducing fecal DCA/LCA, and inhibiting the TGR5/NF-κB pathway (101). Probiotics such as *Bifidobacterium pseudocatenulatum* can increase fecal DCA/LCA, suppressing Th1/Th17 responses via TGR5 in arthritis models (79). Many probiotics exhibit BSH activity, deconjugating primary BAs, which is a critical upstream step influencing the availability of substrates for secondary BA synthesis and modulating FXR signaling (147). These strategies underscore the potential to therapeutically target the microbiota-BA-immune axis, but also highlight the need for personalized approaches considering individual host-microbe-diet interactions and tumor-specific contexts (explored in Section 4.1).

### 3.3 Combination strategies for enhanced anti-tumor efficacy

Modulating the gut microbiota–bile acid (BA) axis offers a promising strategy to enhance the efficacy of existing cancer therapies by reshaping host immunity and TIME. This section highlights its potential synergy with immune checkpoint inhibitors (ICIs), cellular therapies, and conventional chemoradiotherapy.

#### 3.3.1 Synergizing with immune checkpoint inhibitors

The gut microbiota and its bile acid (BA) metabolites have emerged as critical modulators of immune checkpoint inhibitor (ICI) responses. Growing evidence from randomized clinical trials (RCTs) and mechanistic studies indicates that specific microbial signatures—such as enrichment of taxa like *Lachnoclostridium* and *Roseburia inulinivorans*—and associated BA profiles, including elevated levels of ursodeoxycholic acid (UDCA), taurolithocholic acid (TLCA), and chenodeoxycholic acid (CDCA), are consistently correlated with favorable outcomes in hepatocellular carcinoma (HCC), non-small cell lung cancer (NSCLC), and esophageal cancer (68, 119, 148). This highlights the gut microbiota–BA axis as a key regulatory element in modulating immunotherapy efficacy (14, 149).

Mechanistically, these microbial BA metabolites enhance anti-tumor immunity by activating CD8+ T cells, dendritic cells (DCs), and natural killer (NK) cells. Moreover, they may influence novel checkpoint targets such as the NKG2A/HLA-E axis (150). This intricate microbiota–gut microbial metabolite (GMM)–ICI interaction, mediated in part by BA receptors including FXR and TGR5 expressed on immune cells, highlights the expansive role of BAs in shaping immunotherapeutic outcomes (62, 64, 65).

Therapeutic strategies leveraging this microbiota–BA–ICI axis are actively under investigation and have shown encouraging results in both preclinical and early clinical settings (62). Supplementation with beneficial microbes such as *Akkermansia muciniphila* has been demonstrated to improve PD-1 blockade efficacy by modulating BA composition and enhancing T cell infiltration in MAFLD-HCC models (50). Likewise, fecal microbiota transplantation (FMT) and rationally designed microbial consortia (e.g., the MET4 trial investigating a defined multi-strain consortium (151)) are being tested as adjuncts to immunotherapy (14). Notably, live biotherapeutic agents like *Bifidobacterium* CBM588 have shown clinical benefit in RCTs for patients receiving ICI therapy (149).

Dietary modulation of the gut microbiota–BA axis represents another promising approach. Polyphenolic compounds such as castalagin can reshape gut microbial composition, increase taurine-conjugated BA production, and overcome resistance to PD-1 blockade (145).

In addition to microbial interventions, direct pharmacological targeting of BA signaling pathways is also being pursued. Nanodelivery of BA receptor modulators—such as the FXR agonist obeticholic acid (OCA) and the GPBAR1 antagonist 5β-cholanic acid (5β-CA)—enhanced NK, NKT, and CD8+ T cell infiltration and triggered potent anti-tumor responses in liver cancer models (152). Spironolactone, by antagonizing the immunosuppressive secondary BA iso-lithocholic acid (iso-LCA), restored NK cell cytotoxicity and improved ICI efficacy in HCC (44). Even when VDR agonism did not directly enhance therapeutic response, favorable shifts in the gut microbiota and improved survival outcomes were observed, underscoring the BA-responsive nature of VDR (153).

Moreover, innovative modalities such as intestinal low-dose irradiation (ILDR) have demonstrated the ability to synergize with anti–PD-L1 therapy through microbiota- and BA-mediated mechanisms. This includes enrichment of beneficial commensals like *Christensenella minuta* and increased deoxycholic acid (DCA), which can enhance DC maturation and CD8+ T cell priming (60).

Collectively, these diverse findings underscore the translational potential of targeting the microbiota–BA–immune axis to improve ICI outcomes. However, successful clinical implementation will require deeper mechanistic insight, optimized therapeutic strategies, and comprehensive safety profiling (62, 149, 154).

#### 3.3.2 Enhancing cellular therapies through microbiota–BA axis modulation

Although direct clinical evidence is limited, compelling mechanistic data suggest that modulating the gut microbiota–BA axis may augment cellular immunotherapies such as Chimeric Antigen Receptor T (CAR-T) cell therapy. T cell functionality, metabolic fitness, and survival are strongly influenced by the immune and metabolic tone of the TIME, which is shaped in part by BA signaling (62, 150).

Microbiota-modulated BAs have the potential to affect T cell metabolism, including mitochondrial activity, and to modulate exhaustion and immunosuppressive pathways within the TIME (44, 68). Favorable BA modulation may improve CAR-T persistence and effector function while reducing suppressive cell populations such as MDSCs (44, 50, 150). This strategy could also reduce systemic immune-related toxicities, such as cytokine release syndrome (CRS).

Although not yet clinically validated, adjunctive interventions including FMT, probiotics, and BA receptor agonists/antagonists warrant further investigation in the context of cellular therapy, particularly in metabolically dysregulated or immune-resistant tumors (14, 62, 152).

#### 3.3.3 Microbiota–BA interventions as adjuncts to chemotherapy and radiotherapy

Conventional cancer treatments, particularly chemotherapy and radiotherapy, are potent modulators of the gut-liver axis (155). These therapies often cause direct cytotoxicity to the rapidly proliferating intestinal epithelial cells, leading to mucositis and compromising intestinal barrier integrity (156, 157). This damage creates a ripple effect, profoundly altering the gut microbial ecosystem—a phenomenon often referred to as ‘chemotherapy-induced dysbiosis’ (157). Consequently, the metabolic capacity of the microbiota is disrupted, significantly impacting the bile acid (BA) pool (155). For instance, studies have shown that chemotherapeutic agents like melphalan can directly disrupt ileal BA reabsorption (156), while other treatments reduce the abundance of key BA-metabolizing bacteria, such as those within the Clostridiales order. This leads to an impaired conversion of primary to secondary BAs, markedly shifting the overall BA composition. This therapy-induced alteration in the microbiota-BA axis not only contributes to severe treatment-related toxicities, such as diarrhea and enterocolitis, but also reshapes the local and systemic immune landscape, potentially influencing therapeutic outcomes (156, 158). As such, understanding these interactions represents a critical step toward developing strategies that mitigate toxicity and synergize with anti-tumor treatments, including subsequent immunotherapy (155).

Emerging evidence indicates that the gut microbiota–BA axis may also influence responses to conventional therapies such as chemotherapy and radiotherapy. For instance, melphalan-induced disruption of ileal BA reabsorption alters the BA pool and promotes colonic injury through microbiota-mediated mechanisms (156). The efficacy of doxorubicin can be enhanced by probiotic-mediated remodeling of the TIME, and its associated dysbiosis and metabolic alterations—particularly in serum BA profiles—may be mitigated by microbial intervention (159–161).

In radiotherapy, BA-responsive pathways, including those involving the VDR, have been implicated in radioresistance, suggesting that modulating the vitamin D–microbiota–VDR axis could enhance radiosensitivity (162). Moreover, ILDR enhances the efficacy of ICI through microbiota- and BA-mediated mechanisms, including increased DC activation and CD8+ T cell recruitment, potentially driven by DCA enrichment (60).

Together, these findings suggest that targeting the microbiota–BA axis may improve the efficacy and tolerability of chemoradiotherapy. Further studies are required to clarify the complex crosstalk among microbial metabolism, BA signaling, drug response, and immune regulation.

Collectively, these findings highlight a rapidly evolving therapeutic landscape centered on the microbiota–bile acid–immune axis in cancer. **Table 2** compiles these diverse interventions, outlining their mechanisms, representative agents, study stages, and key immune or clinical outcomes.

**Table 2:** Therapeutic strategies targeting the microbiota–bile acid–immune axis in cancer.

Intervention strategy	Specific agent/Method (Type)	Mechanism/Target	Study stage/Model system	Immune & therapeutic outcomes
FXR Modulation	Obeticholic Acid (OCA) (FXR agonist)	FXR activation; ↓ deleterious SBAs and BSH activity; TIME remodeling.	Preclinical (GEAC, HFD-induced dysplasia model; Liver fibrosis model)	Ameliorated dysplasia; Potentially reversed immunosuppressive TIME; Mitigated liver fibrosis.
	Fexaramine D (FexD) (Intestine-specific FXR agonist)	Intestinal FXR activation enhances barrier integrity.	Preclinical (Colitis-associated cancer mouse model)	Reduces colitis-associated cancer progression and M1-like macrophage infiltration.
	Ursodeoxycholic acid (UDCA) (Contextual FXR inhibitor)	FXR inhibition (in specific inflammatory contexts, e.g., RAHR, GVHD).	Preclinical (Radiation-associated hematopoietic recovery model; T cell-driven GVHD mouse model)	Rescues hematopoietic recovery (RAHR); improves survival and reduces IFN-γ expression (GVHD).
TGR5 Modulation	INT-777, DCA (as Gs-biased TGR5 agonists)	Gs-biased TGR5 activation suppresses YAP activity.	Preclinical (NSCLC cell lines; *In vitro*)	Suppresses NSCLC cell proliferation.
	Tumor cell TGR5 signaling (e.g., by DCA/LCA)	TGR5 activation by SBAs (e.g., DCA, LCA) in CRC cells upregulates CCL28 via β-catenin, recruiting Tregs.	Preclinical (CRC mouse model)	Enhances Treg infiltration and immunosuppressive TIME; supports TGR5 as a potential antagonistic target in CRC.
Therapeutic BAs	Ursodeoxycholic acid (UDCA)/Tauroursodeoxycholic acid (TUDCA)	Cytoprotection, Anti-inflammation; Modulates FXR/TGR5.	Preclinical (GVHD mouse model (TUDCA); Colonic inflammation mouse model (UDCA))	TUDCA (GVHD): ↓ Intestinal epithelial apoptosis, ↓ antigen presentation by non-hematopoietic cells. UDCA (Colitis): ↑ M2 macrophage polarization.
	Taurolithocholic acid (TLCA)	Enhances T cell activation and anti-tumor immunity.	Clinical (NSCLC patients on ICIs - Correlational study); Preclinical (*In vitro*/*In vivo*)	Elevated plasma TLCA correlated with improved ICI outcomes; Enhances T cell activation and effector function.
BA Sequestrants	Cholestyramine	Binds and excretes BAs, thereby reducing their enterohepatic recirculation.	Preclinical (Dysbiosis + high soluble fiber-induced HCC mouse model)	Prevented HCC development.
Microbiota-Directed: Enzyme Targeting	Bile Salt Hydrolase (BSH) inhibition	BSH inhibition reduces colonic DCA/LCA production, reduces tumor cell TGR5 activation, and limits Treg recruitment.	Preclinical (CRC mouse model)	Reversed pro-tumorigenic effects; ↓ Treg infiltration.
Microbiota-Directed: FMT	Fecal Microbiota Transplantation (FMT)	Gut microbiota restoration → BA biotransformation normalization.	Clinical (Melanoma patients resistant to ICIs); Preclinical (CDI model)	Overcame anti-PD-1 resistance in melanoma patients; Restored resistance to C. difficile infection.
Microbiota-Directed: Diet/Prebiotics/Probiotics	Dietary Polyphenols (e.g., Castalagin)	Reshapes gut microbiota, increasing taurine-conjugated BAs.	Preclinical (Tumor mouse models + PD-1 blockade)	Circumvented anti-PD-1 resistance; Enhanced anti-tumor activity.
	Inulin (prebiotic) + Rifaximin	Fosters beneficial BA-related bacteria (e.g., Eubacterium) leading to reduced fecal DCA/LCA and inhibition of TGR5/NF-κB signaling.	Preclinical (Colon cancer metastasis mouse model)	Suppressed colon cancer pulmonary metastasis.
	Akkermansia muciniphila (LBP)	Intestinal barrier restoration; BA composition modulation (decreased TCA, DCA levels).	Preclinical (MAFLD-related HCC mouse model + PD-1 blockade)	Enhances PD-1 therapy efficacy; reduces MDSCs and M2 macrophages; increases CD8^+^ T cell infiltration and activation.
	Bifidobacterium CBM588 (LBP)	BA modulation (among other proposed mechanisms)	Clinical (Patients receiving ICI therapy - RCT evidence)	Showed clinical benefit (e.g., improved PFS or response rates).
Combination with ICIs (Pharmacological)	Nanodelivery of FXR agonist (OCA) & GPBAR1 antagonist (5β-CA)	BA receptor signaling modulation in liver.	Preclinical (Liver cancer mouse models)	Enhances infiltration of NK, NKT, and CD8^+^ T cells; elicits potent anti-tumor responses.
	Spironolactone	iso-LCA antagonism.	Preclinical (HCC mouse model + ICI)	Restored NK cell cytotoxicity; Improved ICI efficacy.
Combination with ICIs (Other)	Intestinal Low-Dose Irradiation (ILDR)	Enriches C. minuta, leading to increased DCA levels that enhance DC maturation and CD8^+^ T cell priming.	Clinical (Metastatic cancer patients + anti-PD-L1 therapy - Clinical trial)	Synergistically promotes abscopal ICI effect; increases CD8^+^ T cell infiltration; reduces MDSC accumulation.

This table summarizes therapeutic strategies targeting the microbiota–bile acid–immune axis in cancer. It outlines intervention categories, specific agents or approaches, mechanisms or molecular targets, study stage and model system, and observed immunological and therapeutic outcomes. These strategies and associated evidence reflect findings discussed and referenced in Section 3 of the main text.

BSH, bile salt hydrolase; CRC, colorectal cancer; DCA, deoxycholic acid; FMT, fecal microbiota transplantation; FXR, farnesoid X receptor; GzmB, granzyme B; HCC, hepatocellular carcinoma; ICI, immune checkpoint inhibitor; IFN-γ, interferon gamma; isoLCA, isolithocholic acid; LCA, lithocholic acid; MDSC, myeloid-derived suppressor cell; NK, natural killer (cell); PD-1, programmed cell death protein 1; PD-L1, programmed death-ligand 1; TGR5, Takeda G-protein-coupled receptor 5; TIME, tumor immune microenvironment; TLCA, taurolithocholic acid; Treg, regulatory T cell; UDCA, ursodeoxycholic acid.

### 3.4 Challenges in clinical translation

While the growing understanding of the microbiota–bile acid (BA)–immune axis holds immense therapeutic potential for cancer, translating these insights into clinically effective and safe interventions remains a significant challenge. These hurdles include the inherent complexity and significant inter-individual variability of the axis, challenges in achieving precise and safe interventions, and methodological barriers in clinical trial design and biomarker development, all of which impede rapid ‘bench to bedside’ translation (163, 164).

#### 3.4.1 Inherent axis complexity and inter-individual variability

The microbiota-BA-immune axis is an exceedingly dynamic and multifaceted system. Microbial biotransformation produces a wide range of BA species, where even minor structural changes can lead to differential receptor engagement and functional consequences (163). A critical challenge stems from the mechanistic complexity of BA receptor signaling itself. This is exemplified by the phenomenon of ‘biased agonism’ observed with receptors like TGR5 (GPBAR1) (95, 165). Biased agonism describes how different ligands, upon binding to the same receptor, can preferentially stabilize distinct receptor conformations, thereby selectively activating specific downstream signaling pathways (e.g., Gαs-cAMP vs. β-arrestin pathways) (165). This results in pleiotropic and sometimes contradictory cellular outcomes; for instance, TGR5 activation can be either anti-proliferative or pro-tumorigenic depending on the specific ligand and the pathway engaged (95). This complexity not only makes therapeutic modulation arduous but also presents a sophisticated drug design opportunity: to develop novel, biased TGR5 ligands that selectively promote beneficial anti-inflammatory signaling while avoiding the activation of pro-tumorigenic pathways (95, 165). This intrinsic mechanistic complexity is further compounded by the substantial inter-individual variability in gut microbiota composition, BA profiles, host genetics, and immune responsiveness (63, 166). As highlighted by studies on dietary fiber supplementation, individual responses are highly personalized, influenced by baseline microbial and metabolic states (164, 166). Such heterogeneity complicates the development of universally effective therapies and underscores the need for personalized approaches, which currently face limitations due to an incomplete understanding of these individual determinants (63, 164).

#### 3.4.2 Challenges in intervention precision and safety

Achieving precise modulation of the microbiota–BA axis while ensuring patient safety remains a major challenge across various therapeutic modalities. For pharmacological interventions targeting BA receptors, systemic administration often results in low bioavailability at the intended target (e.g., liver tumors) and potential off-target effects, owing to the widespread distribution of these receptors. The development of nanodelivery systems (152) itself implies challenges with conventional delivery. Moreover, the pleiotropic nature and biased signaling potential of BA receptors (95) raise concerns about unintended consequences, including the inadvertent activation of pro-tumorigenic pathways.

Similarly, microbiota-based interventions are not without risks and complexities. Fecal Microbiota Transplantation (FMT), aimed at restoring beneficial BA metabolism, may yield unpredictable outcomes, such as the increased risk of acute GVHD in some alloHCT recipients, potentially linked to the expansion of conventionally ‘beneficial’ bacteria like *Faecalibacterium*, which may act detrimentally in certain contexts (139). Dietary strategies also warrant caution; for example, fermentable fibers can induce nutrient depletion under inflammatory conditions (167), and high doses of specific fibers like inulin have been associated with inflammatory responses and liver enzyme elevation in humans (166). Probiotic interventions, even those designed to modulate BA profiles via BSH activity, may carry risks such as promoting pathogenic bacterial colonization or increasing levels of pro-carcinogenic secondary BAs (147). These examples underscore that safe and effective microbial manipulation in the complex oncological setting requires a more nuanced understanding than simply “restoring” beneficial elements (63, 139).

#### 3.4.3 Methodological hurdles in clinical translation and biomarker development

The translation of preclinical discoveries into robust clinical applications is significantly impeded by methodological challenges. A major obstacle is the lack of validated surrogate biomarkers that can predict patient response, monitor therapeutic efficacy, or assess the safety of interventions targeting this axis (164, 168). The difficulty in “decoding the microbiota metabolome” (164) and the substantial inter-individual variability (166) make biomarker discovery and validation particularly arduous, compounded by technical limitations in standardized multi-omic data generation and analysis (164).

Furthermore, designing informative clinical trials for these complex interventions is challenging. Challenges include achieving adequate statistical power despite slow disease progression or subtle intervention effects, managing high variability in patient responses, and defining appropriate patient stratification criteria based on complex baseline microbial or metabolic features (63, 168). The translation of findings from rodent models to humans is also often limited by inherent differences in BA metabolism and gut microbiota (63, 147, 163), necessitating rigorous validation in human studies. The current lack of large-scale, integrated cohort studies combining genomic, microbiota, metabolomic, and immune profiling further restricts the establishment of definitive causal links and the development of evidence-based therapeutic strategies (63). Finally, evolving regulatory frameworks for live biotherapeutics and complex nutritional interventions add another layer of translational complexity (63).

## 4 Future perspectives and conclusion

### 4.1 Addressing heterogeneity: tumor type, spatio-temporal dynamics, and host factors

A critical frontier in harnessing the gut microbiota-bile acid (BA) axis for cancer therapy is addressing the profound heterogeneity of its influence on the TIME. Distinct BA metabolic signatures, microbial profiles, and their immunomodulatory consequences are evident across diverse malignancies. For instance, intrahepatic cholangiocarcinoma (ICC) displays unique BA and microbiota features differentiating it from hepatocellular carcinoma (HCC) or cirrhosis (169). Within nonalcoholic steatohepatitis (NASH)-related HCC, the presence of cirrhosis significantly alters primary conjugated BA levels and correlates with specific microbial shifts, such as *Lactobacillus* enrichment, which is linked to disease progression (91).

This variability extends to extra-intestinal sites like breast cancer, where intratumoral BA metabolism scores and microbial compositions are associated with proliferation and survival (170). In pancreatic ductal adenocarcinoma (PDAC), tumor-intrinsic factors, notably KRAS mutation status, and iatrogenic influences like chemotherapy-induced dysbiosis, dramatically reshape BA profiles and modulate the local TIME (160). Furthermore, anatomical location within an organ, such as in colorectal cancer (CRC) where right- versus left-sided tumors harbor distinct microbial and BA environments (171), critically impacts disease progression and the metastatic cascade. Indeed, specific BAs are increasingly implicated in sculpting immunosuppressive pre-metastatic niches, thereby facilitating distant metastasis in cancers like HCC and CRC (12, 172).

The influence of BAs also exhibits significant spatio-temporal dynamics, evolving with tumor progression and responding to therapeutic interventions, as exemplified by chemotherapy effects in PDAC (160). Crucially, host-specific attributes, including sex and genetic predispositions, profoundly modulate BA distribution and its prognostic relevance. In CRC, for example, male patients exhibit distinct right-left colon differences in secondary BA accumulation and Treg cell infiltration that are not mirrored in females (75). Genetic variations affecting BA metabolism, such as those in AKR1D1 which influences iso-LCA production and subsequent NK cell function (as discussed in Section 2.1.2), further underscore this multilayered complexity.

To translate the potential of the microbiota-BA-TIME axis into effective therapies, future research must prioritize the systematic dissection of these context-dependent interactions. This necessitates identifying key microbial effectors and specific BA mediators that drive tumor- and host-specific immune responses. Integrated multi-omics analyses of well-characterized patient cohorts, coupled with rigorous functional validation in advanced preclinical models, will be instrumental. Ultimately, such efforts aim to develop precision interventions targeting the microbiota-BA-immune interplay, tailored to the unique biological landscape of individual cancer types and patient contexts.

### 4.2 Advanced technologies for deciphering the bile acid–microbiota–TIME axis

The intricate interplay between bile acids (BAs), the gut microbiota, and the TIME necessitates sophisticated technological approaches for its comprehensive elucidation. Recent advances in multi-omics platforms have revolutionized our capacity to dissect these complex biological systems. Integrative multi-omics, which encompasses metagenomics (for microbial profiling), transcriptomics, proteomics, and metabolomics, offers a crucial systems-level perspective. Such integrative analyses have revealed correlations between gut microbiota signatures, clinical outcomes, and BA profiles in cancer and related inflammatory conditions (126, 139, 173). Notably, meta-omics (i.e., the combined application of metagenomics, metatranscriptomics, metaproteomics, and metabolomics to microbial communities) provides deep insights into microbiome-mediated immune signaling in pathologies such as colorectal cancer (174).

Advanced metabolomics is central to this effort. Ultra-performance liquid chromatography–tandem mass spectrometry (UPLC-MS/MS) enables precise quantification of structurally diverse BA species (175, 176), facilitating detailed characterization of the “bile acid-ome.” The emerging field of reverse metabolomics further accelerates the discovery of novel bioactive BA derivatives, including previously unannotated microbial metabolites, directly detected in human-derived biospecimens (82).

Beyond bulk analyses, single-cell technologies such as single-cell RNA sequencing (scRNA-seq) and spatial transcriptomics/metabolomics are now providing unprecedented resolution of cellular heterogeneity and spatio-temporal BA signaling within the TIME and associated mucosal tissues (177). These high-resolution tools, when combined with robust computational pipelines, are indispensable for decoding immune-metabolic crosstalk. Advanced bioinformatics approaches, such as enhanced protein–metabolite correlation (178), systems biology modeling (e.g., metabolic model-based integration) (179), and machine learning for BA biomarker discovery in cancer immunotherapy (68), enable the reconstruction of regulatory networks from high-dimensional data (180, 181).

Collectively, these technological advances (15) are deepening our mechanistic understanding of how the gut microbiota and its BA metabolites shape the TIME (182, 183), and are paving the way for novel biomarkers and precision therapeutic strategies targeting this multifaceted axis.

### 4.3 Developing robust biomarkers for precision therapy

The intricate interplay between the gut microbiota, bile acid (BA) metabolism, and the TIME offers a fertile ground for developing robust biomarkers essential for advancing precision oncology. Such biomarkers, derived from microbial signatures, BA profiles, or host responses, have the potential to refine therapeutic selection, improve diagnostic and prognostic accuracy, and guide risk stratification, particularly in gastrointestinal and hepatobiliary cancers.

A critical application lies in predicting response to immune checkpoint inhibitors (ICIs). Specific gut microbial compositions and associated BA metabolites are emerging as key determinants of ICI efficacy. For instance, studies in hepatocellular carcinoma (HCC) have linked favorable ICI outcomes to distinct microbial profiles and elevated levels of certain BAs, notably ursodeoxycholic acid (UDCA) and ursocholic acid (UCA) (119). The presence of *Akkermansia muciniphila*, known to modulate BA metabolism, has also been associated with enhanced PD-1 blockade efficacy in MAFLD-related HCC, suggesting its potential as a predictive biomarker (50). Furthermore, comprehensive metabolomic analyses in non-small cell lung cancer (NSCLC) have identified plasma metabolites, including specific BAs such as taurolithocholic acid (TLCA), as promising predictive biomarkers for ICI response (68).

Beyond predicting treatment response, microbiota-BA axis alterations can serve as diagnostic and prognostic indicators. Integrated analysis of the gut microbiome and host transcriptome has revealed correlations between specific microbial features, BA dysregulation, and clinical outcomes in HBV-related HCC, offering potential prognostic markers (126). In intrahepatic cholangiocarcinoma (ICC), distinct gut microbiota compositions, BA metabolic profiles (e.g., altered primary and secondary BAs), and associated cytokine patterns have been identified, which could aid in differentiating ICC from other liver conditions and predicting prognosis (169). The dysregulation of gut microbial BA metabolism, leading to an accumulation of specific secondary BAs like deoxycholic acid (DCA) and lithocholic acid (LCA), is increasingly recognized for its role in promoting colorectal liver metastasis, suggesting that these BAs or the microbial enzymes producing them could serve as prognostic indicators or markers of metastatic risk (172).

Moreover, this axis may offer biomarkers for early cancer detection and risk assessment. Prediagnostic alterations in circulating bile acid metabolism might signal an increased susceptibility to cancer development, offering a window for preventive strategies (184). For example, associations between ileal juice BA profiles and the presence of colorectal advanced adenomas suggest BAs as potential biomarkers for identifying individuals at high risk for CRC (185). The general dysbiosis of microbial metabolites, beyond just BAs, has also been noted as a potential hallmark in colorectal cancer development (186).

The development of these biomarkers relies heavily on multi-omics technologies and advanced bioinformatic integration to decode the complex “microbiota metabolome” (164, 181). However, translating these discoveries into clinically validated tools requires overcoming challenges related to inter-individual variability, standardization, and the establishment of causality. Future research must focus on rigorous validation in large, diverse cohorts to develop actionable biomarker panels for personalized cancer care.

### 4.4 Unanswered questions and future research directions

Despite significant strides in understanding the interplay between the gut microbiota, bile acid (BA) metabolism, and the TIME, critical questions remain, paving the way for future research.

A paramount challenge is establishing definitive causality beyond correlative observations. While multi-omics studies link microbial signatures, BA profiles, and cancer outcomes (Section 4.3), their precise mechanistic underpinnings are often elusive, a recognized issue in complex diseases (163, 187, 188). Future research must prioritize mechanistic studies using gnotobiotic models, organoid co-cultures, and targeted manipulations to elucidate how specific microbes or their BA metabolites directly modulate immune cell functions (e.g., NK cell cytotoxicity, MDSC expansion, Treg differentiation) within the TIME. For instance, although BAs like DCA and LCA are implicated in colorectal liver metastasis niches, their detailed molecular mechanisms require further investigation (172).

The complexity of BA signaling itself presents another frontier. BAs can interact with multiple receptors (FXR, TGR5, VDR, S1PR2, etc.), and these receptors can exhibit ligand-dependent biased agonism and engage in intricate crosstalk; however, the full functional consequences of these interactions are still being unraveled (2, 127). Key unanswered questions include: How are signals from different BA receptors integrated at the cellular and systemic levels to dictate an overall immune phenotype? What are the dominant BA-receptor pathways in specific tumor types (beyond the emerging data in NSCLC for TGR5 and TLCA) or at different stages of disease, and whether combinatorial signaling through multiple receptors shapes divergent immune outcomes across cancer types? Future directions should include systems biology approaches to map these networks and develop highly selective receptor modulators to dissect specific signaling effects on the TIME.

An exciting, yet unexplored, avenue for future research is the role of BAs in shaping long-term immunological memory. The formation and survival of a robust memory T cell pool are intrinsically linked to specific metabolic programs (189), and as potent metabolic regulators, BAs are perfectly positioned to influence this process. This concept is supported by recent findings where taurolithocholic acid (TLCA) was shown to enhance ICI efficacy by promoting memory CD8^+^ T cell formation, providing a crucial, albeit preliminary, link (68). This opens up critical new research questions: Could other specific BAs drive the metabolic reprogramming essential for memory T cell differentiation? And beyond systemic memory, how does the local BA milieu regulate the function of tissue-resident memory T cells (Trm) within the tumor itself? Answering these questions will be pivotal for developing strategies that “engrave” durable, life-long anti-tumor immunity.

Furthermore, the spatio-temporal dynamics and inherent heterogeneity of the microbiota-BA-immune axis warrant deeper exploration. Microbial composition and BA profiles vary significantly along the gastrointestinal tract and are influenced by host genetics, diet, sex, and concurrent medications, factors contributing to substantial inter-individual variability (164). How these variations impact systemic BA pools and the immune landscape of tumors, particularly those within the hepatobiliary-pancreatic system, and potentially extending to extra-intestinal sites where preliminary associations with BA exposure have been noted (190), needs to be clarified. Advanced *in situ* imaging and sampling techniques, combined with longitudinal multi-omics studies in well-characterized patient cohorts, are essential to capture this dynamic interplay and to identify personalized modulators of this axis. Such studies, as called for in recent reviews (63), should aim to integrate tumor, microbial, and immune data.

Despite growing enthusiasm for therapeutic modulation of the microbiota–BA axis, translating foundational insights into robust clinical applications, including the development of validated biomarkers (Section 4.3), remains a major hurdle. This requires overcoming challenges related to inter-individual variability, methodological standardization, establishing causality, and ensuring the stability and generalizability of biomarkers across diverse populations, as emphasized in studies focusing on decoding the complex microbiota metabolome (164). Similar challenges are also discussed in the context of microbial metabolite dysbiosis (186). For instance, while promising therapeutic strategies targeting this axis are emerging (Sections 3.1-3.3), optimizing their efficacy and safety, and defining appropriate patient populations, requires substantial further work. Key questions include how to design microbial interventions (e.g., engineered probiotics, precision FMT) that reliably and safely reshape the BA pool to favor anti-tumor immunity, and how to overcome challenges of systemic drug delivery and potential off-target effects for BA receptor modulators.

Finally, while much research has focused on the gut-liver axis, further investigation is needed to fully understand the systemic immunomodulatory roles of BAs and their impact on a broader range of cancers beyond primary GI/HB malignancies.

Addressing these unanswered questions through innovative and collaborative research will be pivotal in fully harnessing the therapeutic potential of the microbiota-bile acid-immune axis for cancer prevention, diagnosis, and treatment.

### 4.5 Concluding remarks

The intricate and dynamic interplay between the gut microbiota, bile acid (BA) metabolism, and the TIME represents a paradigm-shifting frontier in cancer research. This review has underscored the fundamental role of this tripartite axis in shaping oncogenesis, tumor progression, and therapeutic responses. We have detailed how the gut microbiota, a vast metabolic organ, orchestrates the transformation of primary BAs into a diverse repertoire of secondary and modified BAs. These microbial BA metabolites, acting as critical signaling integrators, engage a network of host receptors (including FXR, TGR5, VDR, and S1PR2) expressed on immune cells, cancer cells, and stromal cells. This BA-receptor signaling network does not operate in isolation; rather, it translates microbial metabolic cues into a spectrum of cellular responses that collectively sculpt the immunological landscape of the tumor, influencing cellular composition, functional orientation, and the balance between anti-tumor immunity and immune evasion.

A central theme emerging is the frequent association of gut dysbiosis and aberrant BA profiles with the establishment of a pro-inflammatory and immunosuppressive TIME, which facilitates tumor immune evasion and metastasis, particularly in gastrointestinal and hepatobiliary cancers. Specific BAs have been shown to directly influence the differentiation, activation, and effector functions of key innate and adaptive immune cell populations, highlighting a direct mechanistic link between microbial metabolism and anti-tumor immunity.

The growing mechanistic understanding of the microbiota-BA-immune axis offers exciting prospects for novel therapeutic interventions and biomarker development (4, 15). Strategies ranging from dietary modifications, probiotic/prebiotic supplementation, and fecal microbiota transplantation to the pharmacological modulation of BA signaling pathways (e.g., using BA receptor agonists/antagonists or BA sequestrants) are being actively explored, holding potential to reshape the TIME and enhance anti-tumor immunity (2). These approaches hold promise not only as standalone therapies but also as adjuvants to enhance the efficacy of existing treatments, most notably immune checkpoint inhibitors. Furthermore, signatures derived from the microbiota and BA profiles are emerging as potential biomarkers for early cancer detection, risk stratification, prognostic assessment, and prediction of therapeutic response, paving the way for more personalized cancer care.

Despite these advances, significant challenges persist in translating these complex biological insights into routine clinical practice (127, 164). The inherent complexity, inter-individual variability, and dynamic nature of this axis necessitate further research to elucidate precise molecular mechanisms, validate therapeutic targets, and develop robust, clinically applicable biomarkers. Future efforts must focus on rigorous preclinical and clinical studies, leveraging multi-omics technologies and systems biology approaches, to fully unlock the therapeutic potential of modulating this critical regulatory network.

In conclusion, microbial BAs are not passive metabolites but active modulators of anti-tumor immunity. Advancing our understanding of this immunometabolic network will be key to the next generation of precision cancer therapies.
